# Child and adult adiposity and subtype-specific endometrial cancer risk: a multivariable Mendelian randomisation study

**DOI:** 10.1038/s41366-022-01231-y

**Published:** 2022-11-10

**Authors:** Oliver J. Kennedy, Cemsel Bafligil, Tracy A. O’Mara, Xuemin Wang, D. Gareth Evans, Siddhartha Kar, Emma J. Crosbie

**Affiliations:** 1 Division of Cancer Sciences, University of Manchester, Faculty of Biology, Medicine and Health, Saint Mary’s Hospital, Oxford Road, Manchester, UK; 2 Cancer Research Program, QIMR Berghofer Medical Research Institute, Brisbane, QLD Australia; 3 Division of Evolution and Genomic Medicine, University of Manchester, Faculty of Biology, Medicine and Health, St Mary’s Hospital, Manchester, UK; 4 Clinical Genetics Service, Manchester Centre for Genomic Medicine, North West Genomics Laboratory Hub, Manchester University NHS Foundation Trust, Manchester Academic Health Science Centre, Manchester, UK; 5 MRC Integrative Epidemiology Unit, Population Health Sciences, Bristol Medical School, University of Bristol, Bristol, UK; 6 Department of Obstetrics and Gynaecology, Manchester University NHS Foundation Trust, Manchester Academic Health Science Centre, Manchester, UK

**Keywords:** Risk factors, Cancer

## Abstract

Increased adiposity is a known risk factor for endometrial cancer (EC). This study aimed to disentangle the separate causal roles of child and adult adiposity on EC risk in adults, including endometrioid and non-endometrioid histological subtypes using multivariable Mendelian randomisation. These analyses employed genetic associations derived from UK Biobank as proxies for child and adult body size in 12,906 cases and 108,979 controls that participated in the Endometrial Cancer Association Consortium. In multivariable analyses, adult body size increased overall EC (OR 2.30, 95% CI 1.73–3.06) and endometrioid EC risk (OR 2.28, 95% CI 1.65–3.16), while child body size had minimal effect. In contrast, child body size (OR 2.26, 95% CI 1.03–4.99) but not adult body size increased non-endometrioid EC risk. As such, child adiposity has an indirect effect on endometrioid EC risk that is mediated by adult adiposity but has a direct effect on non-endometrioid EC risk that is independent of adult adiposity. These novel findings indicate that interventions targeting adiposity during distinct periods in life have a critical role in preventing subtype-specific EC.

Endometrial cancer (EC) is the second commonest gynaecological cancer worldwide [1]. Incidence will rise by 50% before 2040, mostly due to increasing obesity, the most important risk factor [2, 3]. Childhood obesity is an emerging health crisis and risk factor for multiple cancers [4]. Childhood obesity is associated with EC risk [5] but it is unknown if the association is causal and, if so, whether it represents a direct effect or an indirect effect mediated by adult obesity (since many children with obesity are also affected by obesity as adults). This study aimed to disentangle the importance of adiposity during these two distinct life periods on EC risk, including endometrioid and non-endometrioid histological subtypes.

This study referred to the Strengthening the Reporting of Observational Studies in Epidemiology Using Mendelian Randomization (STROBE-MR) guidelines [6]. Mendelian randomisation (MR) uses genetic variants as instrumental variables (IVs) to identify causal associations between exposures and outcomes [7]. Genetic variants are randomly assigned at conception and unaffected by other factors. Thus, MR has lower risk of bias from confounding and reverse causation. MR is also able to disentangle independent causal effects of multiple related exposures by employing multivariable models [8]. Fig. 1 illustrates the possible causal effects of child and adult body size on EC risk.

**Fig. 1:**
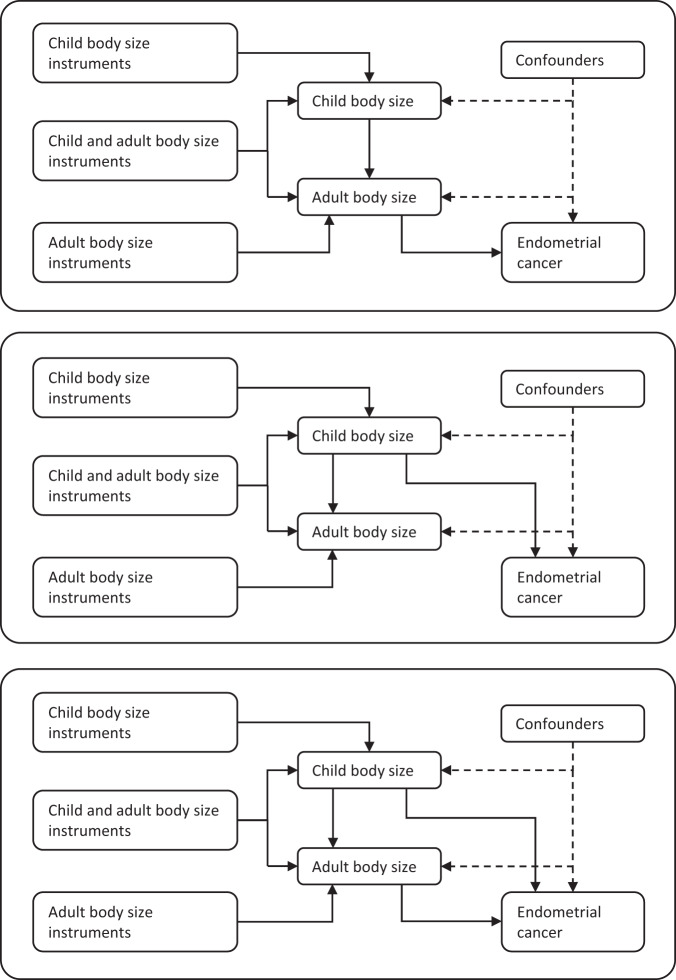
Directed acyclic graphs illustrating the scenarios in which child body size could affect EC risk. Child body size may act indirectly through adult body size (top panel), directly independent of adult body size (middle panel) or both directly and indirectly (bottom panel).

This study employed previously validated sets of single nucleotide polymorphisms (SNPs) associated at genome-wide significance (*p* < 5 × 10^−8^) with child and adult body size (Table S1) [9]. These SNPs were derived from 453,169 participants of European descent aged 40–69, recruited to UK Biobank between 2006 and 2013 [9]. Child body size was based on recollection (at age 10 were you “thinner, plumper, or about average?”), while adult body size was approximated by converting body mass index into a variable with three categories having the same proportions as the child body size variable. Bayesian linear mixed models with age and genotyping array as covariates were used to estimate associations of SNPs with body size. Linkage disequilibrium clumping was performed to remove correlated SNPs. Outcome data for overall, endometrioid and non-endometrioid EC were from the Endometrial Cancer Association Consortium (ECAC) genome-wide association study [10]. ECAC comprised 12,906 cases, including 8758 endometrioid and 1230 non-endometrioid cases, and up to 108,979 controls of European descent. Associations of SNPs with EC risk were estimated using logistic regression with adjustments for principal components. UK Biobank and ECAC both have local and/or national ethics approval and all participants provided written informed consent.

MR relies on three basic assumptions with respect to the IVs to estimate an unconfounded causal association between an exposure and outcome. First, the IVs must be robustly associated with the exposure (i.e. the relevance assumption). Secondly, the IVs must not be associated with confounders of the exposure-outcome association (i.e. the independence assumption). Thirdly, the IVs can be associated with the outcome of interest only through the exposure of interest (i.e. the exclusion restriction assumption).

Univariable and multivariable inverse-variance weighted MR [8] were used to estimate associations of child and adult body size with overall and subtype-specific EC risk. Inverse-variance weighted MR provides reliable estimates if the assumption is met that all SNPs affect the outcome only through the exposure of interest. If SNPs affect the outcome through other pathways (i.e. “horizontal pleiotropy”), inverse-variance weighted MR may still provide reliable estimates provided the net effect of these other pathways on the outcome is zero. However, where the net effect is not zero, bias is introduced from “unbalanced horizontal pleiotropy”. The direction of bias depends whether the overall effect of the invalid SNPs increases or decreases the risk of the outcome.

To investigate possible bias from invalid SNPs, various sensitivity analyses were performed using alternative methods of MR, including MR-Egger [11], weighted median MR [12] and weighted mode MR [13]. Each of these methods employs unique assumptions and provides reliable estimates in the presence of invalid SNPs at the expense of power. MR-Egger is reliable even where all SNPs influence the outcome through pathways not involving the exposure provided these pathways are uncorrelated with the exposure. It also provides a formal test for the presence of unbalanced horizontal pleiotropy. In contrast to MR-Egger, weighted median and weighted mode MR are reliable in the presence of outlying SNPs but only allow for a proportion of the SNPs to be invalid (in weighted median this proportion is < 50% while in weighted mode it is assume that the mode of the individual SNP-level effects represents the true effect). Further details of these methods are summarised in a recent review [14]. Associations were estimated as odds ratios (OR) with 95% confidence intervals (CI) per change in body size category. Analyses were performed in R (Version 4.1.1) using the TwoSampleMR [15] and MendelianRandomization [16] packages, while the Metafor package [17] was used for constructing forest plots. Potential bias from sample overlap was assessed using the method of Burgess et al. [18] as implemented in a web application available at https://sb452.shinyapps.io/overlap/.

There were 166 and 288 SNPs associated with child and adult body size, respectively, available in the ECAC dataset. In univariable analyses (Fig. 2, Table S2), child and adult body size were associated with overall EC (child OR = 1.86, 95% CI = 1.52–2.28; adult OR = 2.41, 95% CI = 2.00–2.91), endometrioid EC (child OR = 2.09, 95% CI = 1.66–2.65; adult OR = 2.56, 95% CI = 2.07–3.17) and non-endometrioid EC (child OR = 2.23, 95% CI = 1.32–3.79; adult OR = 1.67, 95% CI = 1.04–2.67). In multivariable analyses (Table S3), adult body size was associated with overall EC (OR = 2.30, 95% CI = 1.73–3.06) and endometrioid EC (OR = 2.28, 95% CI = 1.65–3.16), but not non-endometrioid EC (1.06, 0.52–2.17). In contrast, child body size was associated with non-endometrioid EC (OR = 2.26, 95% CI = 1.03–4.99), but its associations with overall EC (OR = 1.11, 95% CI = 0.81-1.52) and endometrioid EC (OR = 1.25, 95% CI = 0.87–1.79) included unity. The results from MR-Egger were similar in direction and magnitude and provided no statistical evidence of bias from pleiotropy (Tables S2–S5). The estimated type 1 error rate remained at 0.05 after taking into account overlapping samples.

**Fig. 2:**
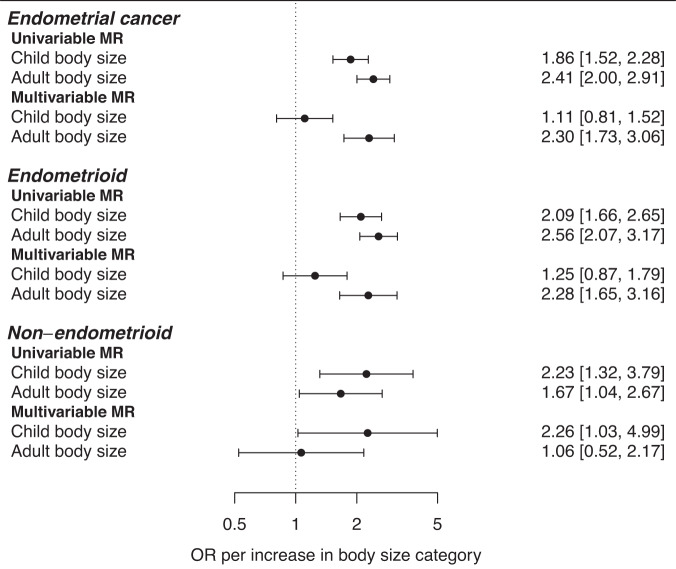
Forest plot showing the univariable and multivariable MR results for child and adult body size on endometrial cancer risk. Estimates are shown as odds ratios and 95% confidence intervals.

This is the first study to disentangle the causal associations of child and adult adiposity with the risks of overall and subtype-specific EC. When both are considered together, only adult adiposity is a strong risk factor for endometrioid EC. Given endometrioid EC accounts for around 90% of cases [19], this highlights weight loss in the transition from childhood to adulthood as a potentially effective strategy for reducing the population level burden of EC by breaking the frequently observed link between child and adult adiposity [20].

This study also newly identifies a link between child adiposity and non-endometrioid EC that is independent of adult adiposity. While rarer, non-endometrioid EC is more aggressive, routinely requires cytotoxic treatment and has a lower 5-year survival [21]. Our results suggest that the higher rates of non-endometrioid EC observed among adults with obesity are explained by these adults likely being children with obesity previously. Given rapidly rising rates of childhood obesity, this association warrants urgent investigation.

The strong association in this study between adult adiposity and endometrioid EC risk is similar to previous studies [3, 22], though a direct comparison of effect sizes is not possible given differing units of exposure (i.e. body size vs. body mass index). However, we establish for the first time that this association is independent of childhood adiposity. Endometrioid tumours are sensitive to oestrogen, of which adipocytes are a major source in adults with obesity [23] but not children [24]. This may explain the lack of a direct effect of child body size on endometrioid EC risk.

The mechanism underlying the association between child body size and non-endometrioid EC is unclear. The uterus may be more susceptible to damage from obesity-induced inflammation during childhood. A pro-inflammatory environment during rapid peri-pubescent endometrial thickening may also be important. Anti-tumorigenic natural killer cells are functionally deficient in children with obesity and this may persist into adulthood [25].

Strengths of this study included large sample sizes, use of validated SNPs as instruments for body size [9], and the quality of case-control ascertainment. MR was less susceptible to bias compared to traditional observational methods. Moreover, multivariable MR offered a powerful approach to separate causal effects of child and adult body size on EC risk.

Weaknesses included the ascertainment of child body size, which was susceptible to recall bias. UK Biobank is a healthy volunteer population including males and females, and so not necessarily representative of a general female population. There was some overlap of the exposure and outcome samples, which could have led to MR model overfitting, though the estimated effect on the type 1 error rate was negligible.

In summary, adult adiposity is a strong risk factor for endometrioid EC, but child adiposity has only a minimal independent effect (after accounting for adult adiposity). Therefore, weight loss in children with obesity transitioning into adulthood may be an effective strategy to reduce population level burden of EC, since most cases are endometrioid. However, non-endometrioid EC may be associated with child adiposity independently of adult adiposity, and the prevention of child adiposity altogether may be the better strategy for this aggressive subtype. Further work is needed to confirm these findings and elucidate the poorly understood underlying biological mechanisms.

## Supplementary information

Supplementary material

## Data Availability

The data used in this study are publicly available.
