# Supplementary material for: Child and adult adiposity and subtype-specific endometrial cancer risk: a multivariable Mendelian randomisation study
Source: Int J Obes (Lond). 2022 Nov 10;47(1):87–90. doi: 10.1038/s41366-022-01231-y (PMC9834041; doi:10.1038/s41366-022-01231-y)
Supplement: Supplementary file 1 — Supplementary material [file 41366_2022_1231_MOESM1_ESM.docx]

Child and adult adiposity and subtype-specific endometrial cancer risk: a multivariable Mendelian randomisation study – supplementary material

Oliver J. Kennedy^1^, Cemsel Bafligil^1^, Tracy A. O'Mara^2^, Xuemin Wang^2^, D. Gareth Evans^3,4^, Siddhartha Kar^5*^, Emma J. Crosbie^1,6*^

1- Division of Cancer Sciences, University of Manchester, Faculty of Biology, Medicine and Health, Saint Mary’s Hospital, Oxford Road, Manchester, UK

2- Cancer Research Program, QIMR Berghofer Medical Research Institute, Brisbane, Queensland, Australia

3- Division of Evolution and Genomic Medicine, University of Manchester, Faculty of Biology, Medicine and Health, St Mary's Hospital, Manchester, UK

4- Clinical Genetics Service, Manchester Centre for Genomic Medicine, North West Genomics Laboratory Hub, Manchester University NHS Foundation Trust, Manchester Academic Health Science Centre, Manchester, UK

5- MRC Integrative Epidemiology Unit, Population Health Sciences, Bristol Medical School, University of Bristol, UK

6- Department of Obstetrics and Gynaecology, Manchester University NHS Foundation Trust, Manchester Academic Health Science Centre, Manchester, UK

* These authors contributed equally to this work

**Supplementary Table 1: SNP associations with child body size, adult body size and endometrial cancer used in this Mendelian randomisation study**

| **SNP** | **Effect allele** | **Other allele** | **Beta adult body size** | **Standard error adult body size** | **Beta child body size** | **Standard error child body size** | **Beta EC** | **Standard error EC** | **Beta endometrioid** | **Standard error endometrioid** | **Beta non-endometrioid** | **Standard error non-endometrioid** |
| --- | --- | --- | --- | --- | --- | --- | --- | --- | --- | --- | --- | --- |
| rs1000471 | C | T | 0.00091 | 0.00169 | -0.00951 | 0.001725 | -0.0172 | 0.019233 | -0.02828 | 0.022603 | 0.038928 | 0.05672 |
| rs10095724 | G | A | 0.002955 | 0.00143 | 0.009371 | 0.001461 | 0.002393 | 0.015973 | 0.008096 | 0.018775 | 0.033177 | 0.045971 |
| rs10111937 | C | T | -0.0049 | 0.001496 | -0.00837 | 0.001528 | 0.002596 | 0.016631 | -0.01798 | 0.019534 | 0.053995 | 0.048434 |
| rs10116891 | G | A | -0.00994 | 0.002286 | -0.0136 | 0.002335 | -0.00118 | 0.026298 | 0.013688 | 0.031277 | -0.02921 | 0.077453 |
| rs10133279 | C | T | -0.00483 | 0.001391 | -0.00896 | 0.00142 | -0.00115 | 0.016164 | 0.001564 | 0.019071 | -0.03436 | 0.046559 |
| rs10146997 | A | G | -0.016 | 0.001651 | -0.00869 | 0.001686 | -0.04076 | 0.01828 | -0.05125 | 0.021372 | 0.114191 | 0.05441 |
| rs10169594 | T | C | -0.00788 | 0.001427 | -0.00045 | 0.001459 | -0.00591 | 0.016185 | -0.00399 | 0.019136 | -0.00655 | 0.047116 |
| rs10174253 | A | C | -0.01121 | 0.001532 | -0.00308 | 0.001567 | -0.02174 | 0.017285 | -0.01662 | 0.020331 | -0.04423 | 0.050236 |
| rs10182458 | A | G | -0.01995 | 0.001369 | -0.03591 | 0.0014 | -0.00874 | 0.015637 | -0.01325 | 0.018479 | -0.12231 | 0.045473 |
| rs10204994 | G | A | 0.010113 | 0.001628 | 0.000702 | 0.001665 | 0.008281 | 0.018417 | 0.006499 | 0.021666 | 0.042585 | 0.053731 |
| rs10234366 | G | A | -0.00175 | 0.002243 | -0.01396 | 0.002292 | -0.02409 | 0.024563 | -0.02018 | 0.028902 | -0.05154 | 0.069116 |
| rs1040046 | C | A | -0.01126 | 0.001921 | -0.00125 | 0.001963 | -0.01057 | 0.02145 | -0.01227 | 0.025275 | -0.03623 | 0.061492 |
| rs10404726 | C | T | 0.012293 | 0.001378 | 0.006556 | 0.001407 | 0.030866 | 0.01575 | 0.025297 | 0.018644 | 0.049542 | 0.045618 |
| rs10423928 | T | A | 0.020764 | 0.001732 | 0.011795 | 0.001769 | 0.03359 | 0.019541 | 0.021499 | 0.022965 | 0.042085 | 0.057324 |
| rs10499014 | C | G | 0.010046 | 0.001556 | -0.00066 | 0.00159 | 0.02011 | 0.017583 | 0.042925 | 0.020747 | -0.04073 | 0.049718 |
| rs10505836 | A | C | -0.01214 | 0.001992 | -0.00308 | 0.002034 | 0.020166 | 0.021637 | 0.019766 | 0.025358 | 0.066123 | 0.061232 |
| rs10760277 | C | T | -0.00887 | 0.001414 | -0.00168 | 0.001444 | -0.00501 | 0.016139 | -0.00455 | 0.019082 | 0.034811 | 0.047241 |
| rs10779835 | T | C | 0.00801 | 0.001406 | 0.000337 | 0.001437 | 0.002236 | 0.016281 | 0.007819 | 0.019308 | -0.0665 | 0.048177 |
| rs10790809 | A | G | -0.00657 | 0.00138 | -0.00967 | 0.00141 | -0.00547 | 0.017554 | -0.02206 | 0.021803 | 0.014397 | 0.055565 |
| rs10791113 | A | G | -0.00857 | 0.001375 | -0.00396 | 0.001405 | 0.002365 | 0.015298 | -0.00575 | 0.018619 | -0.02368 | 0.046103 |
| rs10799778 | T | G | 0.012582 | 0.001839 | 0.007051 | 0.00188 | 0.01348 | 0.020831 | 0.00405 | 0.024642 | 0.071056 | 0.058753 |
| rs10805383 | G | A | -0.01047 | 0.001374 | -0.00157 | 0.001404 | -0.01638 | 0.015157 | -0.01473 | 0.017791 | -0.01574 | 0.043349 |
| rs10823504 | G | A | 0.000902 | 0.002812 | 0.015862 | 0.002873 | 0.031606 | 0.032048 | 0.041357 | 0.037874 | 0.064959 | 0.092727 |
| rs10823826 | C | T | -0.00898 | 0.001589 | -0.00256 | 0.001623 | 0.016188 | 0.017811 | 0.01515 | 0.020996 | 0.049219 | 0.051954 |
| rs10860295 | T | C | -0.00181 | 0.001381 | -0.00852 | 0.00141 | 0.014777 | 0.0154 | 0.017398 | 0.018115 | 0.06122 | 0.044466 |
| rs10887571 | C | T | -0.00682 | 0.001389 | -0.00798 | 0.00142 | -0.02521 | 0.016066 | -0.0127 | 0.018923 | -0.13102 | 0.046368 |
| rs10927006 | T | C | 0.011993 | 0.001954 | 0.000884 | 0.001997 | 0.014103 | 0.021637 | -0.01327 | 0.025316 | 0.136225 | 0.064912 |
| rs11000993 | T | C | -0.01381 | 0.002079 | -0.00634 | 0.002124 | 0.034674 | 0.02349 | 0.048747 | 0.027865 | -0.01861 | 0.066771 |
| rs11047138 | C | G | -0.01438 | 0.002629 | -0.00508 | 0.002685 | -0.00538 | 0.028945 | 0.022894 | 0.034241 | -0.02028 | 0.08409 |
| rs11079849 | C | T | 0.012234 | 0.001464 | 0.001417 | 0.001495 | 0.012136 | 0.016712 | 0.003865 | 0.019776 | -0.04301 | 0.051136 |
| rs11134679 | A | G | -0.0124 | 0.00148 | -0.0086 | 0.001512 | 0.016243 | 0.016468 | 0.015826 | 0.019442 | 0.03868 | 0.047884 |
| rs11150462 | T | A | 0.008444 | 0.001425 | 0.003096 | 0.001455 | -0.01719 | 0.016323 | -0.0125 | 0.019352 | -0.06552 | 0.048034 |
| rs11150745 | A | G | 0.013795 | 0.001476 | 0.012151 | 0.001507 | -0.02533 | 0.016646 | -0.02561 | 0.019642 | -0.05069 | 0.050814 |
| rs11205303 | T | C | 0.001236 | 0.001394 | 0.009582 | 0.001425 | 0.013104 | 0.015685 | 0.03019 | 0.018502 | 0.035899 | 0.044527 |
| rs11209943 | A | G | -0.01408 | 0.001397 | -0.01834 | 0.001428 | -0.00788 | 0.015634 | -0.00433 | 0.018377 | -0.01576 | 0.044776 |
| rs11215403 | G | A | 0.00591 | 0.0016 | 0.013237 | 0.001634 | -0.00928 | 0.018101 | -0.02979 | 0.021986 | 0.010898 | 0.055137 |
| rs112253053 | T | A | 0.013771 | 0.00187 | 0.010143 | 0.001909 | 0.016035 | 0.020943 | 0.040914 | 0.024787 | -0.10897 | 0.058174 |
| rs112380819 | G | A | -0.01369 | 0.002258 | -0.00105 | 0.002309 | -0.01837 | 0.025081 | -0.03618 | 0.029355 | -0.08236 | 0.072228 |
| rs11256627 | G | A | -0.00095 | 0.001514 | -0.00888 | 0.001547 | 0.030257 | 0.016859 | 0.036376 | 0.019811 | 0.063738 | 0.048172 |
| rs112875651 | G | A | -0.00835 | 0.001423 | -0.00479 | 0.001454 | 0.020764 | 0.016053 | 0.01125 | 0.018929 | 0.03607 | 0.046085 |
| rs113079574 | C | T | 0.009652 | 0.001746 | -0.00099 | 0.001785 | 0.01482 | 0.019214 | 0.012899 | 0.022528 | -0.01685 | 0.054456 |
| rs113132247 | G | A | -0.01233 | 0.001911 | -0.0055 | 0.001952 | -0.0198 | 0.021623 | -0.0409 | 0.025445 | 0.012962 | 0.063265 |
| rs113230003 | G | A | 0.012616 | 0.001577 | 0.006999 | 0.00161 | 0.031358 | 0.017939 | 0.037233 | 0.021112 | 0.059041 | 0.051573 |
| rs114593013 | A | G | 0.019718 | 0.002945 | 0.011553 | 0.003011 | 0.004126 | 0.033873 | -0.01468 | 0.039873 | -0.11096 | 0.096005 |
| rs114728753 | A | C | -0.00895 | 0.001466 | -0.00132 | 0.001499 | -0.00027 | 0.016366 | -0.00643 | 0.019149 | -0.07447 | 0.046844 |
| rs11496125 | C | T | -0.01092 | 0.001395 | -0.00854 | 0.001425 | -0.0015 | 0.015535 | -0.00325 | 0.018316 | 0.001895 | 0.044503 |
| rs11525873 | T | C | 0.014829 | 0.002313 | 0.017501 | 0.002363 | 0.035292 | 0.026874 | 0.054879 | 0.03194 | -0.16428 | 0.074426 |
| rs115319174 | G | C | -0.01131 | 0.002969 | -0.04234 | 0.003036 | -0.08719 | 0.033918 | -0.05759 | 0.040309 | -0.21899 | 0.09233 |
| rs115359679 | C | A | -0.00538 | 0.002799 | -0.01643 | 0.002859 | -0.03086 | 0.036345 | -0.04063 | 0.043401 | 0.023209 | 0.105142 |
| rs115778101 | T | C | 0.017768 | 0.003219 | 0.006923 | 0.003291 | -0.04349 | 0.039329 | -0.01978 | 0.046923 | -0.09779 | 0.111031 |
| rs115866895 | A | G | 0.012019 | 0.001566 | 0.005603 | 0.001601 | -0.01418 | 0.026663 | 0.026309 | 0.035132 | -0.16241 | 0.072184 |
| rs115903965 | G | A | 0.001858 | 0.004443 | -0.02551 | 0.004544 | 0.047965 | 0.077406 | 0.015435 | 0.097502 | -0.39651 | 0.201661 |
| rs11642090 | T | C | -0.00728 | 0.001428 | -0.01171 | 0.001459 | 0.011787 | 0.015994 | 0.016479 | 0.01885 | -0.00461 | 0.04611 |
| rs11655704 | T | C | 0.002442 | 0.001473 | 0.008272 | 0.001505 | 0.040419 | 0.016552 | 0.032094 | 0.019474 | 0.067515 | 0.050923 |
| rs1167311 | G | A | 0.012253 | 0.001479 | 0.004773 | 0.001512 | 0.000784 | 0.016202 | 0.00341 | 0.01906 | 0.027033 | 0.046181 |
| rs11691869 | C | A | 0.011231 | 0.001427 | -0.00036 | 0.001459 | -0.02616 | 0.016331 | -0.02108 | 0.019297 | -0.07972 | 0.047055 |
| rs11765062 | T | C | 0.007516 | 0.001375 | 0.000937 | 0.001405 | 0.008461 | 0.015403 | 0.009988 | 0.018145 | -0.02541 | 0.044259 |
| rs1177279 | A | G | 0.002064 | 0.00153 | 0.008831 | 0.001564 | 0.025976 | 0.017042 | 0.030403 | 0.020027 | 0.015548 | 0.049061 |
| rs11782074 | G | T | -0.00972 | 0.001433 | 0.000824 | 0.001464 | -0.00088 | 0.015726 | -0.0213 | 0.018407 | 0.050192 | 0.044888 |
| rs117903946 | G | A | -0.00978 | 0.003866 | -0.03225 | 0.003949 | 0.044675 | 0.043956 | 0.094937 | 0.052301 | 0.040044 | 0.127562 |
| rs117911387 | G | A | -0.00368 | 0.003261 | -0.02458 | 0.003331 | -0.0483 | 0.040601 | -0.11355 | 0.047537 | -0.07299 | 0.119121 |
| rs11891707 | T | C | 0.000436 | 0.002009 | 0.012105 | 0.002054 | -0.02918 | 0.023336 | -0.02675 | 0.027608 | -0.04776 | 0.067157 |
| rs11976084 | C | T | -0.00857 | 0.001519 | -0.00661 | 0.001552 | -0.02791 | 0.017792 | -0.02278 | 0.020995 | -0.0963 | 0.051496 |
| rs12031634 | G | A | 0.008806 | 0.001505 | 0.003603 | 0.001538 | 0.009406 | 0.017546 | 0.017592 | 0.02082 | 0.014032 | 0.051674 |
| rs12033257 | A | G | 0.009803 | 0.001419 | -0.00076 | 0.001451 | 0.023449 | 0.016352 | 0.04674 | 0.019357 | -0.02125 | 0.047398 |
| rs12037905 | C | T | 0.007927 | 0.001388 | 0.000749 | 0.001419 | -0.04669 | 0.015577 | -0.04809 | 0.018354 | -0.03493 | 0.04463 |
| rs12042908 | A | G | 0.010443 | 0.00138 | 0.02746 | 0.001411 | -0.00381 | 0.015265 | 0.004731 | 0.017947 | -0.00692 | 0.043581 |
| rs12072739 | A | G | -0.01159 | 0.001643 | -0.00089 | 0.00168 | -0.00027 | 0.018254 | -0.01341 | 0.021427 | -0.0105 | 0.052545 |
| rs12140153 | G | T | 0.021447 | 0.002401 | 0.021828 | 0.002455 | 0.018399 | 0.027724 | 0.018277 | 0.032709 | 0.014421 | 0.08012 |
| rs12149660 | G | A | 0.016218 | 0.002161 | 0.004008 | 0.002207 | 0.004415 | 0.024439 | 0.00164 | 0.028863 | 0.006641 | 0.071771 |
| rs12213441 | C | T | -0.01051 | 0.001672 | -0.00385 | 0.001709 | 0.004668 | 0.019975 | 0.004194 | 0.023667 | -0.02752 | 0.057233 |
| rs12214497 | G | T | 0.005635 | 0.001442 | 0.010517 | 0.001473 | -0.02886 | 0.016026 | -0.02948 | 0.018856 | -0.05124 | 0.04612 |
| rs1222216 | C | T | 0.012152 | 0.001637 | 0.005085 | 0.001672 | 0.024485 | 0.01838 | 0.018796 | 0.022432 | 0.106009 | 0.05661 |
| rs12253527 | G | A | -0.01361 | 0.00147 | -9.6E-06 | 0.001501 | -0.03771 | 0.016057 | -0.01948 | 0.01887 | -0.09488 | 0.045505 |
| rs1229984 | T | C | -0.02304 | 0.004164 | 0.00059 | 0.004256 | 0.092009 | 0.045574 | 0.078681 | 0.053936 | 0.394208 | 0.120368 |
| rs12357890 | A | G | -0.01219 | 0.001388 | -0.00153 | 0.001418 | -0.01276 | 0.015885 | -0.00879 | 0.018785 | -0.01876 | 0.04635 |
| rs12427047 | C | T | 0.01091 | 0.001599 | 0.001403 | 0.001633 | 0.021465 | 0.01748 | 0.017806 | 0.020435 | -0.01848 | 0.04978 |
| rs12429545 | G | A | -0.02003 | 0.002062 | -0.02063 | 0.002106 | -0.03477 | 0.023002 | -0.03872 | 0.02706 | -0.01921 | 0.06628 |
| rs12450028 | C | T | 0.006858 | 0.001443 | 0.011564 | 0.001473 | 0.014494 | 0.016708 | 0.011962 | 0.019829 | 0.061366 | 0.052255 |
| rs12462975 | G | A | -0.01148 | 0.00147 | -0.00515 | 0.001501 | -0.00101 | 0.016327 | -0.0038 | 0.019191 | -0.01175 | 0.046801 |
| rs12484438 | T | C | 0.012887 | 0.001451 | 0.01178 | 0.001481 | 0.035371 | 0.016058 | 0.035009 | 0.018872 | -0.02072 | 0.045702 |
| rs12517187 | C | T | -0.00825 | 0.001388 | -0.00451 | 0.001418 | 0.007377 | 0.015591 | -0.00776 | 0.018301 | 0.026217 | 0.044526 |
| rs12641981 | C | T | -0.01867 | 0.001385 | -0.0222 | 0.001415 | -0.02991 | 0.015303 | -0.0428 | 0.017998 | 0.043902 | 0.043955 |
| rs12681792 | C | A | -0.00956 | 0.001746 | -0.00069 | 0.001783 | -0.05793 | 0.019793 | -0.06796 | 0.023352 | -0.08384 | 0.057016 |
| rs12713889 | T | C | 0.005705 | 0.001454 | 0.011691 | 0.001487 | 0.018436 | 0.016097 | 0.031873 | 0.018952 | 0.034518 | 0.046209 |
| rs12748436 | C | G | -0.00565 | 0.002584 | -0.01561 | 0.002642 | -0.02044 | 0.029051 | -0.01897 | 0.033907 | -0.11658 | 0.082594 |
| rs12788343 | T | C | -0.00965 | 0.001393 | -0.0051 | 0.001423 | -0.02506 | 0.016083 | -0.01545 | 0.01983 | -0.0477 | 0.049818 |
| rs12798028 | C | T | -0.01503 | 0.001392 | -0.01456 | 0.001422 | -0.01591 | 0.015485 | -0.03356 | 0.018823 | 0.042854 | 0.047091 |
| rs12821683 | G | C | -0.01118 | 0.002013 | -0.00532 | 0.002055 | -0.00231 | 0.022944 | 0.005864 | 0.027076 | -0.05372 | 0.064912 |
| rs1286138 | T | G | -0.009 | 0.001467 | -0.00544 | 0.001498 | -0.00364 | 0.016238 | 0.00599 | 0.019109 | -0.0516 | 0.046953 |
| rs12883788 | C | T | -0.01243 | 0.001382 | -0.01004 | 0.001411 | -0.02279 | 0.015543 | -0.03775 | 0.018303 | -0.00492 | 0.044858 |
| rs12941038 | C | T | -0.00475 | 0.001634 | -0.00954 | 0.001669 | -0.0171 | 0.018264 | 0.003933 | 0.021566 | -0.0522 | 0.054783 |
| rs12951079 | G | A | 0.010788 | 0.001405 | 0.007424 | 0.001435 | 0.012615 | 0.016286 | 0.020496 | 0.019338 | -0.07657 | 0.051076 |
| rs1296328 | A | C | 0.011738 | 0.001387 | 0.00804 | 0.001418 | 0.022365 | 0.015609 | 0.022197 | 0.01841 | 0.010817 | 0.045211 |
| rs1296685 | A | G | -0.00949 | 0.001697 | -0.00609 | 0.001732 | -0.01994 | 0.018969 | -0.02968 | 0.022205 | -0.03084 | 0.053778 |
| rs12971645 | G | A | 0.008594 | 0.001543 | 0.000343 | 0.001576 | 0.039926 | 0.017458 | 0.053231 | 0.020622 | 0.001106 | 0.049891 |
| rs12992672 | G | A | -0.03595 | 0.001811 | -0.04335 | 0.001852 | -0.02851 | 0.020561 | -0.03506 | 0.024221 | 0.033118 | 0.058394 |
| rs13047416 | C | G | 0.008657 | 0.001422 | 0.012342 | 0.001451 | 0.015149 | 0.015953 | 0.026494 | 0.018797 | 0.025243 | 0.04587 |
| rs13107325 | C | T | -0.02871 | 0.002606 | -0.02151 | 0.002663 | 0.007724 | 0.030194 | 0.033312 | 0.036229 | -0.07674 | 0.087043 |
| rs13174863 | A | G | -0.01362 | 0.001944 | -0.00403 | 0.001987 | -0.02224 | 0.022944 | -0.01296 | 0.027214 | -0.02796 | 0.066932 |
| rs13186637 | T | C | 0.008683 | 0.001441 | -0.00065 | 0.001473 | -0.00205 | 0.016457 | -0.01813 | 0.01923 | -0.03393 | 0.047441 |
| rs1320251 | C | T | 0.012393 | 0.001383 | -0.00037 | 0.001412 | -0.01745 | 0.01539 | -0.02735 | 0.018081 | -0.00283 | 0.046781 |
| rs1320903 | G | A | -0.01462 | 0.001469 | -0.01001 | 0.001502 | -0.0019 | 0.016714 | -0.01332 | 0.019703 | 0.007063 | 0.048159 |
| rs13218383 | C | G | 0.009318 | 0.001452 | 0.004225 | 0.001483 | -7.5E-05 | 0.016187 | 0.010998 | 0.019079 | -0.03937 | 0.04655 |
| rs13254613 | A | C | 0.005649 | 0.001444 | -0.01257 | 0.001475 | 0.014802 | 0.016549 | 0.023972 | 0.01959 | -0.02905 | 0.04868 |
| rs13275517 | T | C | -0.00815 | 0.001386 | -0.00081 | 0.001416 | 0.011353 | 0.015539 | 0.010856 | 0.018239 | 0.033258 | 0.044464 |
| rs13292699 | A | C | 0.012536 | 0.001386 | -0.00147 | 0.001416 | 0.020276 | 0.015396 | 0.016105 | 0.018081 | 0.005745 | 0.043692 |
| rs1333010 | G | A | 0.006531 | 0.001408 | 0.012068 | 0.001438 | 0.011396 | 0.016215 | -0.00778 | 0.019165 | 0.04679 | 0.047045 |
| rs13427822 | A | G | 0.009449 | 0.001556 | -0.00058 | 0.001591 | 0.007573 | 0.01753 | 0.004546 | 0.020609 | 0.054529 | 0.050961 |
| rs1342831 | T | C | -0.00659 | 0.002952 | -0.02248 | 0.003016 | -0.02484 | 0.033211 | -0.04772 | 0.039211 | -0.11584 | 0.09508 |
| rs1369159 | C | T | 0.007875 | 0.001396 | 0.002632 | 0.001425 | 0.033889 | 0.016079 | 0.027421 | 0.019011 | 0.044184 | 0.046232 |
| rs1384660 | G | A | 0.007457 | 0.001763 | 0.015805 | 0.001803 | 0.006999 | 0.019776 | 0.016539 | 0.023311 | 0.097839 | 0.058452 |
| rs1402989 | C | T | -0.00403 | 0.001371 | -0.00779 | 0.001402 | 0.010459 | 0.015199 | 0.002854 | 0.01782 | 0.077315 | 0.043629 |
| rs1411432 | A | C | -0.01244 | 0.001768 | 0.001494 | 0.001806 | 0.007961 | 0.02031 | 0.006303 | 0.024055 | 0.026968 | 0.059738 |
| rs1422067 | C | T | 0.008702 | 0.00161 | 0.01088 | 0.001645 | -0.02011 | 0.018936 | -0.01478 | 0.022546 | -0.03742 | 0.055963 |
| rs142315514 | C | A | -0.02139 | 0.00379 | -0.01386 | 0.003875 | 0.006413 | 0.043998 | 0.005032 | 0.0525 | -0.00605 | 0.128121 |
| rs1423534 | G | A | 0.007709 | 0.001394 | 0.007145 | 0.001425 | -0.00369 | 0.015661 | -0.01592 | 0.018486 | 0.058984 | 0.045583 |
| rs1436348 | A | G | -0.00966 | 0.001389 | -0.00446 | 0.00142 | -0.02224 | 0.015619 | -0.02175 | 0.018432 | -0.03405 | 0.044954 |
| rs143662847 | C | T | 0.019318 | 0.003534 | 0.009315 | 0.00361 | 0.062555 | 0.043373 | 0.087077 | 0.051889 | -0.03296 | 0.127434 |
| rs1451533 | G | A | -0.01097 | 0.001546 | -0.00384 | 0.001581 | -0.01974 | 0.017626 | -0.01195 | 0.020858 | -0.07074 | 0.050961 |
| rs1452991 | G | A | -0.00577 | 0.001427 | -0.01006 | 0.001458 | -0.00345 | 0.015848 | -0.01113 | 0.018666 | -0.03242 | 0.044968 |
| rs1458156 | C | T | -0.00929 | 0.001373 | -0.00119 | 0.001402 | 0.009439 | 0.015157 | 0.016255 | 0.017807 | 0.021775 | 0.043448 |
| rs146910503 | G | A | 0.014782 | 0.004914 | 0.033417 | 0.005025 | -0.12635 | 0.057909 | -0.05935 | 0.069195 | -0.48347 | 0.148775 |
| rs1476698 | A | G | 0.001385 | 0.001418 | 0.008222 | 0.00145 | -0.01179 | 0.015741 | -0.0146 | 0.018517 | -0.05689 | 0.044934 |
| rs1477290 | T | C | -0.01994 | 0.00201 | -0.00815 | 0.002054 | -0.01254 | 0.021908 | -0.03221 | 0.025654 | 0.038692 | 0.064869 |
| rs147730268 | G | T | 0.022832 | 0.002485 | -0.00115 | 0.002538 | 0.05538 | 0.027922 | 0.046062 | 0.032916 | 0.101499 | 0.084368 |
| rs149457 | C | T | 0.015032 | 0.00183 | 0.001984 | 0.00187 | 0.044301 | 0.020437 | 0.051675 | 0.024129 | -0.00514 | 0.05732 |
| rs149778057 | A | C | 0.009045 | 0.001535 | 0.000437 | 0.001567 | 0.003375 | 0.01815 | 0.017709 | 0.021402 | 0.006875 | 0.054269 |
| rs151252883 | T | G | -0.00922 | 0.001442 | -0.00191 | 0.001474 | 0.014199 | 0.016739 | 0.00308 | 0.019657 | 0.042612 | 0.049883 |
| rs1568488 | G | C | -0.01103 | 0.001406 | -0.00386 | 0.001438 | 0.013851 | 0.015908 | 0.017446 | 0.018765 | 0.083659 | 0.045571 |
| rs1576655 | A | C | -0.01135 | 0.001427 | -0.01185 | 0.001458 | 0.010138 | 0.016221 | 0.00565 | 0.019231 | -0.00978 | 0.047532 |
| rs1582931 | G | A | 0.009657 | 0.001384 | 0.005305 | 0.001415 | -0.01408 | 0.01534 | -0.02394 | 0.018034 | 0.001212 | 0.043798 |
| rs1633418 | T | C | 0.007921 | 0.00141 | 0.004885 | 0.001439 | 0.019096 | 0.015498 | 0.014047 | 0.018217 | -0.02481 | 0.044502 |
| rs1696057 | T | C | 0.003914 | 0.001447 | 0.008312 | 0.001477 | 0.002971 | 0.016383 | -0.02121 | 0.019368 | 0.014023 | 0.047328 |
| rs16996644 | C | G | -0.0106 | 0.002068 | -0.01935 | 0.00211 | -0.00086 | 0.023809 | -0.01936 | 0.028213 | -0.03646 | 0.070024 |
| rs17029006 | C | T | 0.010397 | 0.001553 | 0.00442 | 0.001589 | -0.02352 | 0.016995 | -0.02487 | 0.02001 | -0.07443 | 0.048224 |
| rs17399739 | A | G | -0.01775 | 0.002712 | -0.02096 | 0.002771 | -0.05154 | 0.030214 | -0.0447 | 0.035356 | 0.060576 | 0.089228 |
| rs17619860 | T | C | -0.0104 | 0.00186 | -0.00567 | 0.0019 | -0.05437 | 0.020217 | -0.04225 | 0.023652 | -0.05753 | 0.057138 |
| rs17716502 | C | T | 0.016236 | 0.001714 | 0.004708 | 0.001751 | 0.032722 | 0.018984 | 0.008739 | 0.022288 | -0.00631 | 0.05389 |
| rs1775255 | G | T | -0.00946 | 0.001375 | -0.01335 | 0.001405 | 0.008783 | 0.01517 | 0.013497 | 0.017998 | 0.003302 | 0.043932 |
| rs17770336 | C | T | -0.01552 | 0.001464 | -0.00384 | 0.001496 | -0.03488 | 0.016268 | -0.01142 | 0.019135 | -0.03271 | 0.046336 |
| rs17773370 | G | A | -0.01589 | 0.002761 | -0.01186 | 0.002819 | -5.4E-05 | 0.03706 | -0.07684 | 0.044265 | 0.050599 | 0.113895 |
| rs1778830 | G | A | -0.00991 | 0.001427 | -0.00374 | 0.001459 | -0.02753 | 0.01575 | -0.0218 | 0.018574 | -0.10205 | 0.044745 |
| rs1788808 | A | G | 0.01274 | 0.001375 | 0.008811 | 0.001404 | 0.022516 | 0.015366 | 0.027609 | 0.018093 | 0.022798 | 0.044085 |
| rs1805123 | T | G | 0.011047 | 0.001593 | 0.005839 | 0.001628 | -0.00065 | 0.017836 | -0.00448 | 0.020966 | -0.00086 | 0.051025 |
| rs1840660 | G | A | -0.00995 | 0.001415 | 0.001436 | 0.001446 | 0.020548 | 0.015809 | 0.033024 | 0.018613 | -0.02927 | 0.045086 |
| rs1852006 | G | A | 0.008831 | 0.00143 | 0.008773 | 0.001461 | -0.00252 | 0.016024 | -0.01051 | 0.018844 | -0.00046 | 0.046315 |
| rs1899689 | C | T | -0.00774 | 0.001405 | -0.0031 | 0.001435 | -0.00699 | 0.015994 | -0.02338 | 0.018909 | 0.041561 | 0.047047 |
| rs1933437 | G | A | 0.007247 | 0.001415 | 0.014162 | 0.001446 | 0.013662 | 0.015601 | 0.008459 | 0.018336 | 0.050945 | 0.044464 |
| rs200744777 | T | G | -0.00938 | 0.001393 | -0.01213 | 0.001422 | -0.02936 | 0.015673 | -0.04532 | 0.018306 | -0.01507 | 0.045765 |
| rs2034963 | G | C | 0.005004 | 0.001447 | 0.011087 | 0.00148 | 0.005749 | 0.016244 | 0.001416 | 0.019126 | -0.05803 | 0.046769 |
| rs2035806 | G | A | 0.009549 | 0.001386 | 0.002563 | 0.001416 | 0.00196 | 0.015382 | 0.019436 | 0.018072 | -0.0491 | 0.04416 |
| rs2051559 | T | C | -0.01386 | 0.002029 | -0.01031 | 0.002073 | -0.02682 | 0.022443 | -0.02354 | 0.026419 | -0.09999 | 0.062784 |
| rs2083323 | G | A | -0.01041 | 0.0018 | -0.00503 | 0.001838 | -0.01732 | 0.019719 | -0.02759 | 0.023111 | 0.031424 | 0.057833 |
| rs2114210 | G | A | -0.00986 | 0.001454 | -0.00302 | 0.001485 | 0.023063 | 0.016302 | 0.0215 | 0.0192 | 0.050376 | 0.047222 |
| rs212517 | T | A | 0.001562 | 0.0014 | 0.008753 | 0.001431 | 0.014134 | 0.015575 | 0.027559 | 0.018319 | -0.00524 | 0.044925 |
| rs215634 | A | G | 0.010434 | 0.001411 | 0.00344 | 0.001442 | 0.000945 | 0.015768 | 0.007178 | 0.018631 | -0.03378 | 0.045434 |
| rs217672 | A | C | -0.01181 | 0.001547 | 0.001019 | 0.00158 | 0.047828 | 0.017999 | 0.057037 | 0.021352 | 0.051462 | 0.052636 |
| rs2187642 | A | C | -0.00158 | 0.001415 | -0.01118 | 0.001445 | -0.01074 | 0.015931 | -0.03005 | 0.018727 | -0.01244 | 0.046016 |
| rs2207894 | C | T | 0.007601 | 0.001745 | 0.014354 | 0.001781 | 0.009945 | 0.019755 | 0.034208 | 0.02341 | -0.04718 | 0.056357 |
| rs2229330 | T | G | -0.00347 | 0.002635 | -0.01974 | 0.002694 | -0.0256 | 0.033941 | -0.01865 | 0.040633 | -0.10053 | 0.0979 |
| rs2234458 | C | T | 0.012819 | 0.001426 | 0.012577 | 0.001457 | -0.00561 | 0.015941 | -0.00558 | 0.019425 | -0.02922 | 0.048265 |
| rs2237025 | T | C | 0.010243 | 0.001391 | -0.00029 | 0.001422 | 0.032635 | 0.015914 | 0.038653 | 0.018795 | -0.00278 | 0.046255 |
| rs2238435 | C | G | -0.01415 | 0.001412 | -0.01648 | 0.001442 | -0.01383 | 0.015939 | -0.01436 | 0.018795 | -0.01076 | 0.046241 |
| rs2253310 | C | G | -0.01056 | 0.001416 | -0.00514 | 0.001447 | -0.00523 | 0.015634 | -0.01176 | 0.018381 | -0.00404 | 0.044747 |
| rs2268762 | A | G | -0.00439 | 0.001404 | -0.00799 | 0.001436 | -0.02294 | 0.016128 | -0.03655 | 0.019133 | 0.046179 | 0.046927 |
| rs2281148 | T | C | -0.00555 | 0.001586 | -0.00979 | 0.001619 | 0.003325 | 0.017852 | 0.001861 | 0.021073 | -0.05421 | 0.050481 |
| rs2289379 | C | T | 0.009759 | 0.001408 | 0.00457 | 0.001438 | 0.039297 | 0.015989 | 0.040461 | 0.018857 | -0.0237 | 0.04597 |
| rs2307111 | T | C | 0.016755 | 0.001403 | 0.008736 | 0.001433 | 0.005259 | 0.015551 | -0.00569 | 0.018267 | -0.01861 | 0.044353 |
| rs2343681 | G | A | -0.01236 | 0.001678 | -0.00451 | 0.001715 | -0.0264 | 0.018569 | -0.02874 | 0.021882 | 0.003823 | 0.052906 |
| rs236660 | T | C | -0.01447 | 0.00144 | -0.00127 | 0.001471 | -0.00885 | 0.017143 | -0.01369 | 0.020031 | -0.07178 | 0.045437 |
| rs2425856 | A | G | 0.008422 | 0.001381 | 0.003478 | 0.00141 | 0.020976 | 0.015638 | 0.013844 | 0.01846 | 0.065691 | 0.045342 |
| rs2433733 | G | A | 0.010504 | 0.001464 | 0.000691 | 0.001497 | -0.02943 | 0.016167 | -0.02886 | 0.018996 | -0.05287 | 0.046536 |
| rs2450444 | G | A | 0.008074 | 0.001438 | 0.00524 | 0.001469 | -0.00539 | 0.016008 | -0.00587 | 0.018826 | -0.04779 | 0.045623 |
| rs2576135 | T | A | 0.013167 | 0.002387 | 0.00121 | 0.002438 | -0.01192 | 0.027022 | -0.02875 | 0.032039 | 0.104114 | 0.075401 |
| rs2594994 | T | A | 0.002107 | 0.00179 | 0.015449 | 0.00183 | -0.01755 | 0.020067 | -0.01116 | 0.023567 | -0.09789 | 0.058763 |
| rs2616143 | G | A | 0.008848 | 0.001474 | 0.000515 | 0.001506 | -0.00219 | 0.016249 | -0.00105 | 0.019108 | 0.013994 | 0.046588 |
| rs2678204 | T | G | -0.01502 | 0.001445 | -0.00575 | 0.001477 | -0.03104 | 0.016073 | -0.02653 | 0.01892 | -0.01249 | 0.046589 |
| rs2722406 | C | T | -0.00431 | 0.001522 | -0.01219 | 0.001555 | -0.00804 | 0.017035 | -0.01356 | 0.020085 | -0.01347 | 0.049554 |
| rs2725371 | A | G | 0.010038 | 0.001497 | -0.00346 | 0.001529 | 0.025112 | 0.016657 | 0.025601 | 0.019621 | 0.028915 | 0.048181 |
| rs2735556 | T | C | 0.012947 | 0.002148 | 0.017187 | 0.002197 | -0.00785 | 0.024526 | -0.02618 | 0.028765 | -0.06582 | 0.068693 |
| rs2767486 | A | G | -0.00136 | 0.001705 | -0.01537 | 0.001743 | -0.01617 | 0.019256 | -0.01133 | 0.02268 | 0.058704 | 0.056317 |
| rs28350 | A | G | 0.012636 | 0.001791 | 0.009697 | 0.001832 | 0.02271 | 0.020612 | 0.023815 | 0.024399 | 0.089617 | 0.060005 |
| rs28462076 | A | G | 0.009393 | 0.00162 | 0.00624 | 0.001656 | 0.007697 | 0.01823 | 0.006162 | 0.02149 | 0.002403 | 0.052192 |
| rs28711392 | T | C | 0.011779 | 0.001434 | 0.005761 | 0.001465 | 0.013772 | 0.015711 | 0.008406 | 0.019087 | 0.026097 | 0.04762 |
| rs2875762 | G | C | -0.01104 | 0.001604 | -0.00517 | 0.001639 | 0.008929 | 0.018478 | 0.022576 | 0.021903 | -0.00246 | 0.053598 |
| rs2968973 | C | T | 0.008114 | 0.001431 | 0.009927 | 0.001462 | 0.011758 | 0.015934 | 0.017153 | 0.018753 | 0.010787 | 0.045448 |
| rs2999158 | T | C | -0.00326 | 0.00145 | 0.008869 | 0.001482 | -0.00852 | 0.016365 | -0.01096 | 0.01929 | 0.002031 | 0.047036 |
| rs3172332 | T | C | 0.002694 | 0.001424 | 0.00801 | 0.001456 | 0.011737 | 0.016092 | 0.005786 | 0.018979 | 0.005805 | 0.046972 |
| rs329651 | G | T | -0.01109 | 0.001736 | -0.00717 | 0.001773 | -0.02493 | 0.019802 | -0.01991 | 0.024378 | 0.067726 | 0.059167 |
| rs34260097 | T | G | -0.00236 | 0.001643 | -0.01782 | 0.001679 | 0.000121 | 0.018413 | 0.006603 | 0.021736 | -0.03484 | 0.05261 |
| rs34292685 | C | T | 0.012723 | 0.00186 | 0.005312 | 0.0019 | 0.062597 | 0.021294 | 0.068907 | 0.026091 | 0.011692 | 0.064233 |
| rs34298980 | T | C | 0.013257 | 0.001444 | 0.005885 | 0.001476 | -0.00051 | 0.017214 | 0.005922 | 0.020396 | -0.00778 | 0.050264 |
| rs34517439 | C | A | -0.0247 | 0.002117 | -0.01377 | 0.002165 | -0.04726 | 0.024334 | -0.04866 | 0.028674 | -0.05229 | 0.069659 |
| rs34778589 | A | C | -0.01434 | 0.0025 | -0.01032 | 0.002551 | -0.02349 | 0.028256 | -0.00933 | 0.033504 | 0.021055 | 0.082347 |
| rs34811474 | G | A | 0.016596 | 0.001627 | 0.010212 | 0.001663 | -0.01233 | 0.020846 | -0.0316 | 0.024698 | 0.07982 | 0.061102 |
| rs34994596 | T | C | 0.011151 | 0.001502 | 0.004775 | 0.001533 | 0.033821 | 0.016977 | 0.04472 | 0.020085 | -0.01869 | 0.048195 |
| rs35193668 | C | T | 0.01047 | 0.001431 | 0.005678 | 0.001461 | 0.013011 | 0.015795 | 0.007896 | 0.018575 | 0.022775 | 0.044991 |
| rs35221880 | T | C | 0.008573 | 0.001554 | 0.004725 | 0.001587 | 0.044073 | 0.017214 | 0.036185 | 0.02024 | 0.081454 | 0.048578 |
| rs35809007 | G | A | 0.010592 | 0.001427 | 0.004336 | 0.00146 | 0.029804 | 0.01607 | 0.019391 | 0.018934 | 0.032089 | 0.046349 |
| rs35918296 | C | T | 0.010176 | 0.001394 | 0.016651 | 0.001424 | 0.020918 | 0.015318 | 0.034527 | 0.018054 | -0.03556 | 0.043827 |
| rs35957544 | G | T | 0.012679 | 0.00139 | 0.001334 | 0.00142 | -0.00761 | 0.015613 | -7.7E-05 | 0.018419 | -0.06399 | 0.045504 |
| rs36007635 | G | A | 0.013712 | 0.00199 | 0.002646 | 0.002034 | -0.01385 | 0.022341 | -0.01872 | 0.026312 | 0.002654 | 0.064233 |
| rs3737992 | G | A | 0.013732 | 0.001825 | 0.003074 | 0.001866 | -0.00465 | 0.019913 | 0.020434 | 0.023458 | -0.04987 | 0.05677 |
| rs3810291 | G | A | -0.01579 | 0.001465 | -0.01474 | 0.001495 | -0.01462 | 0.016347 | -0.00465 | 0.019238 | -0.05364 | 0.047246 |
| rs3814883 | C | T | -0.01476 | 0.001376 | -0.00609 | 0.001406 | -0.019 | 0.015504 | -0.03184 | 0.018215 | 0.067179 | 0.044425 |
| rs3815156 | A | G | -0.00529 | 0.001806 | -0.01031 | 0.001844 | -0.02738 | 0.019861 | -0.03201 | 0.023305 | -0.00097 | 0.060908 |
| rs3817428 | C | G | -0.00319 | 0.001556 | -0.01075 | 0.001588 | -0.00638 | 0.018575 | -0.01051 | 0.022003 | -0.01506 | 0.054887 |
| rs3823674 | C | T | 0.007657 | 0.001389 | 0.004842 | 0.001419 | 0.04153 | 0.015511 | 0.033953 | 0.018253 | 0.065 | 0.044935 |
| rs3902951 | T | G | -0.01052 | 0.001632 | -0.00473 | 0.001666 | -0.02568 | 0.017729 | -0.04071 | 0.020762 | -0.04254 | 0.050654 |
| rs3931548 | C | A | -0.01051 | 0.001439 | -0.00541 | 0.001469 | -0.01172 | 0.016296 | -0.01842 | 0.019199 | 0.040442 | 0.047496 |
| rs396354 | T | C | 0.010398 | 0.00152 | 0.001363 | 0.001555 | 0.033546 | 0.016933 | 0.027495 | 0.019921 | -0.00349 | 0.048738 |
| rs39674 | C | G | -0.00871 | 0.001498 | -0.00073 | 0.00153 | 0.002177 | 0.01649 | 0.008659 | 0.019439 | 0.026053 | 0.047216 |
| rs403694 | C | T | -0.01218 | 0.001383 | -0.00186 | 0.001411 | 0.005931 | 0.01529 | 0.011655 | 0.017951 | -0.01044 | 0.044055 |
| rs409696 | G | A | 0.011422 | 0.001387 | 0.001546 | 0.001418 | 0.015292 | 0.01596 | 0.000525 | 0.018885 | 0.011087 | 0.047039 |
| rs41310284 | C | A | 0.017741 | 0.002291 | 0.020604 | 0.002341 | 0.058082 | 0.027038 | 0.056074 | 0.032189 | -0.01669 | 0.07697 |
| rs4148155 | A | G | 0.014349 | 0.002158 | 0.006416 | 0.002205 | 0.005835 | 0.024244 | 0.007264 | 0.02865 | -0.0041 | 0.069862 |
| rs429358 | T | C | 0.016069 | 0.001902 | -0.00345 | 0.001942 | 0.067093 | 0.022323 | 0.077307 | 0.026463 | 0.154548 | 0.066334 |
| rs4419475 | A | T | -0.0081 | 0.001395 | 0.001156 | 0.001426 | -0.04975 | 0.01551 | -0.05363 | 0.018244 | -0.0613 | 0.044329 |
| rs4432271 | C | T | -0.01156 | 0.002063 | -0.01753 | 0.002108 | -0.03348 | 0.022562 | -0.04635 | 0.026659 | -0.06146 | 0.065637 |
| rs4482463 | C | A | 0.018863 | 0.002574 | 0.009467 | 0.002632 | 0.004555 | 0.029009 | -0.02681 | 0.034387 | 0.06727 | 0.081843 |
| rs4545941 | T | C | -0.00212 | 0.001864 | -0.01088 | 0.001903 | -0.00565 | 0.024055 | 0.002234 | 0.028817 | 0.022827 | 0.069483 |
| rs4572029 | A | G | -0.00072 | 0.001713 | 0.011108 | 0.00175 | -0.00412 | 0.01859 | -0.00906 | 0.021809 | 0.118663 | 0.054984 |
| rs4575195 | C | A | 0.009594 | 0.00148 | 0.004427 | 0.001512 | 0.039578 | 0.016507 | 0.044452 | 0.019478 | -0.01251 | 0.046847 |
| rs4648450 | C | A | 0.009801 | 0.00138 | 0.004355 | 0.00141 | 0.027438 | 0.015456 | 0.030961 | 0.018187 | -0.0546 | 0.044037 |
| rs4658403 | C | T | 0.013642 | 0.00184 | 9.35E-05 | 0.001881 | 0.002035 | 0.020643 | -0.00207 | 0.024305 | 0.025957 | 0.058872 |
| rs4671328 | T | G | 0.01317 | 0.001386 | 0.005622 | 0.001417 | 0.005008 | 0.015324 | -0.00238 | 0.017994 | 0.033731 | 0.043866 |
| rs4672338 | C | T | -0.00835 | 0.001448 | -0.0046 | 0.001481 | -0.02432 | 0.016153 | -0.01964 | 0.019026 | -0.02475 | 0.046438 |
| rs4688359 | C | T | 0.010469 | 0.001417 | 0.013048 | 0.001449 | -0.02138 | 0.015929 | -0.01044 | 0.018774 | -0.0454 | 0.045944 |
| rs473837 | G | T | 0.008631 | 0.001436 | -0.00101 | 0.001467 | -0.00975 | 0.016273 | -0.02452 | 0.019231 | 0.009053 | 0.047514 |
| rs4739558 | A | G | 0.008097 | 0.001401 | 0.008295 | 0.001431 | -0.00162 | 0.015627 | -0.00402 | 0.018407 | 0.046534 | 0.04466 |
| rs4744246 | A | G | 0.000944 | 0.001448 | -0.01576 | 0.001479 | -0.0057 | 0.016026 | -0.01432 | 0.018856 | -0.02735 | 0.046015 |
| rs4764949 | A | G | 0.011935 | 0.001465 | 0.008012 | 0.001496 | 0.000331 | 0.016261 | 0.010312 | 0.019056 | 0.065032 | 0.046745 |
| rs4783789 | T | C | 0.001654 | 0.001648 | 0.009583 | 0.001683 | -0.00874 | 0.018443 | -0.00941 | 0.02173 | -0.00995 | 0.052792 |
| rs4790292 | C | A | 0.016329 | 0.001911 | 0.007818 | 0.001951 | -0.0104 | 0.021893 | -0.03535 | 0.025631 | 0.05474 | 0.07084 |
| rs4806814 | G | A | 0.012651 | 0.001904 | -0.0021 | 0.001944 | -0.00045 | 0.021486 | -0.01341 | 0.025223 | -0.00537 | 0.062907 |
| rs4911382 | C | T | -0.00832 | 0.001397 | -0.00223 | 0.001426 | 0.022285 | 0.015498 | 0.011289 | 0.018201 | 0.092612 | 0.044114 |
| rs4916229 | C | G | -0.01513 | 0.002333 | 0.000377 | 0.002385 | -0.04246 | 0.026174 | -0.03258 | 0.030687 | -0.05517 | 0.075744 |
| rs4958568 | G | A | 0.004806 | 0.001533 | 0.009956 | 0.001567 | 0.010188 | 0.017076 | 0.008619 | 0.020077 | -0.01474 | 0.048387 |
| rs4962725 | T | C | -0.00997 | 0.001387 | -0.00139 | 0.001417 | -0.02167 | 0.015355 | -0.00742 | 0.018074 | -0.03102 | 0.043891 |
| rs512121 | T | C | 0.010571 | 0.001749 | 0.009305 | 0.001786 | -0.00691 | 0.019456 | 0.000967 | 0.023019 | -0.03864 | 0.055773 |
| rs537508 | G | C | -0.00777 | 0.001396 | -0.00326 | 0.001427 | -0.00317 | 0.015614 | 0.008554 | 0.018396 | 0.033272 | 0.044883 |
| rs543874 | A | G | -0.03012 | 0.001694 | -0.04712 | 0.001732 | -0.00346 | 0.018986 | -0.00781 | 0.022284 | 0.005724 | 0.054678 |
| rs544957562 | A | T | 0.012393 | 0.002023 | 0.005739 | 0.002066 | 0.010683 | 0.023536 | 0.039262 | 0.02749 | -0.08012 | 0.066602 |
| rs55658481 | G | A | -0.00897 | 0.001444 | -0.00816 | 0.001477 | 0.005212 | 0.016395 | -0.0023 | 0.01932 | -0.02165 | 0.046887 |
| rs55726687 | G | A | -0.01376 | 0.001683 | -0.0145 | 0.001718 | -0.0128 | 0.01866 | -0.02624 | 0.021905 | -0.02909 | 0.053349 |
| rs55838622 | A | C | -0.0099 | 0.001644 | -0.00673 | 0.00168 | -0.01412 | 0.018766 | -0.02336 | 0.022079 | -0.00395 | 0.054373 |
| rs55880046 | T | G | 0.016863 | 0.001963 | 0.029855 | 0.002005 | 0.065389 | 0.021973 | 0.07909 | 0.025979 | 0.054522 | 0.062589 |
| rs55896564 | G | A | 0.011697 | 0.001382 | 0.009113 | 0.001412 | 0.008803 | 0.019081 | 0.008658 | 0.02249 | -0.04717 | 0.064568 |
| rs559231 | G | T | -0.00952 | 0.001412 | -0.0091 | 0.001441 | -0.01116 | 0.015681 | -0.01531 | 0.018433 | -0.0194 | 0.044623 |
| rs55931203 | C | T | -0.01256 | 0.001778 | -0.00192 | 0.001815 | -0.08928 | 0.019742 | -0.10674 | 0.023124 | -0.02042 | 0.060387 |
| rs56094641 | A | G | -0.04652 | 0.001397 | -0.04726 | 0.001427 | -0.04819 | 0.015348 | -0.06274 | 0.018029 | -0.01123 | 0.044085 |
| rs56803094 | A | G | 0.009577 | 0.001645 | 0.005942 | 0.001679 | -0.0055 | 0.018334 | -0.00894 | 0.021526 | -0.04544 | 0.052604 |
| rs575840515 | A | G | -0.00901 | 0.001521 | -0.0019 | 0.001555 | -0.02311 | 0.017556 | -0.02194 | 0.020671 | 0.023651 | 0.052466 |
| rs57590313 | C | A | -0.01017 | 0.001788 | -0.00698 | 0.001828 | -0.04257 | 0.020609 | -0.05093 | 0.024389 | 0.010533 | 0.060571 |
| rs57636386 | T | C | 0.024702 | 0.002485 | 0.023501 | 0.002537 | 0.003463 | 0.027407 | -0.00938 | 0.032195 | 0.020852 | 0.079452 |
| rs57654548 | C | A | 0.011629 | 0.002122 | 0.002016 | 0.00217 | -0.0086 | 0.023332 | -0.00978 | 0.027436 | -0.02409 | 0.066417 |
| rs58084604 | C | T | -0.03519 | 0.001622 | -0.03318 | 0.001656 | -0.01273 | 0.017855 | -0.01405 | 0.02095 | -0.05272 | 0.050885 |
| rs59227842 | A | G | -0.01524 | 0.001494 | -0.00057 | 0.001526 | 0.008026 | 0.016414 | 0.02126 | 0.020087 | 0.006803 | 0.050274 |
| rs59714050 | T | A | -0.01937 | 0.002753 | -0.02284 | 0.002815 | -0.03861 | 0.031107 | -0.01834 | 0.036692 | -0.01718 | 0.089006 |
| rs601338 | G | A | 0.000917 | 0.001371 | 0.009381 | 0.0014 | -0.00828 | 0.015287 | -0.00471 | 0.017992 | -0.00656 | 0.043792 |
| rs6029180 | A | G | -0.00819 | 0.001479 | -0.00457 | 0.001509 | -0.02642 | 0.016462 | -0.04573 | 0.019343 | -0.00793 | 0.047628 |
| rs6030803 | T | C | 0.012927 | 0.002074 | 0.0043 | 0.002116 | 0.025814 | 0.023775 | 0.020312 | 0.028097 | 0.017801 | 0.068912 |
| rs6050446 | A | G | -0.02642 | 0.003895 | -0.0067 | 0.003975 | -0.03634 | 0.045298 | -0.02166 | 0.05419 | 0.001438 | 0.134771 |
| rs60644673 | G | T | -0.00406 | 0.00174 | 0.010029 | 0.001777 | -0.03774 | 0.019399 | -0.04214 | 0.02277 | -0.06836 | 0.055557 |
| rs6075658 | T | C | 0.008489 | 0.001376 | 0.002426 | 0.001404 | -0.0155 | 0.01521 | -0.01946 | 0.017861 | -0.06568 | 0.043344 |
| rs61217499 | G | C | 0.012759 | 0.001672 | 0.001597 | 0.001707 | 0.002095 | 0.019474 | 0.006734 | 0.023144 | -0.09182 | 0.056448 |
| rs61754230 | C | T | -0.02738 | 0.004928 | -0.00981 | 0.005032 | -0.05765 | 0.083988 | -0.11518 | 0.104816 | -0.14892 | 0.230835 |
| rs61813324 | C | T | -0.0183 | 0.002027 | -0.00534 | 0.002073 | -0.00876 | 0.024333 | -0.00505 | 0.02873 | -0.02777 | 0.070303 |
| rs61903695 | A | G | -0.01132 | 0.001575 | -0.00187 | 0.001609 | -0.04995 | 0.017703 | -0.05077 | 0.021635 | 0.057002 | 0.055685 |
| rs61937656 | G | A | 0.005067 | 0.001644 | 0.011723 | 0.001678 | 0.037639 | 0.018402 | 0.03953 | 0.02172 | 0.004107 | 0.053612 |
| rs61971082 | T | G | -0.01013 | 0.001523 | 0.000162 | 0.001556 | -0.00353 | 0.016987 | -0.00278 | 0.019982 | 0.006807 | 0.049175 |
| rs61978655 | G | A | -0.01769 | 0.003572 | -0.03393 | 0.003648 | -0.04967 | 0.041235 | -0.0366 | 0.048964 | -0.01555 | 0.124204 |
| rs61985411 | T | A | -0.01653 | 0.002759 | -0.00147 | 0.002817 | -0.02599 | 0.028798 | -0.01257 | 0.033721 | -0.17233 | 0.079404 |
| rs61992671 | A | G | 0.00988 | 0.001435 | 0.007579 | 0.001465 | 0.016188 | 0.017423 | 0.01077 | 0.020686 | 0.056308 | 0.048844 |
| rs62048187 | G | C | -0.00601 | 0.001501 | -0.00839 | 0.001532 | 0.025667 | 0.017476 | 0.03537 | 0.020706 | 0.000802 | 0.050588 |
| rs62106258 | T | C | 0.059139 | 0.003182 | 0.080205 | 0.003254 | 0.124988 | 0.043487 | 0.078662 | 0.051884 | 0.229719 | 0.132785 |
| rs62134189 | A | G | -0.00182 | 0.002273 | 0.014197 | 0.002325 | -0.0051 | 0.026195 | 0.005929 | 0.030789 | 0.032015 | 0.077603 |
| rs62277889 | C | T | 0.010595 | 0.001582 | 0.006407 | 0.001617 | 0.011694 | 0.018305 | 0.019986 | 0.021694 | 0.027104 | 0.052039 |
| rs62379271 | T | G | -0.00781 | 0.001392 | -0.00322 | 0.001422 | -0.0153 | 0.015926 | -0.00521 | 0.018848 | -0.09302 | 0.046385 |
| rs62425398 | C | A | -0.00912 | 0.002235 | -0.01541 | 0.002284 | -0.01278 | 0.026388 | -0.01103 | 0.031316 | -0.05727 | 0.07653 |
| rs62621197 | C | T | -0.0065 | 0.003769 | -0.02353 | 0.003848 | 0.024595 | 0.054645 | 0.031441 | 0.066068 | -0.0833 | 0.150608 |
| rs6265 | C | T | 0.024542 | 0.001753 | 0.011703 | 0.001791 | 0.039411 | 0.019482 | 0.033288 | 0.023757 | 0.113551 | 0.05998 |
| rs6449532 | C | T | 0.006977 | 0.001426 | 0.011053 | 0.001457 | 0.010262 | 0.015989 | -0.00561 | 0.018783 | 0.006911 | 0.046282 |
| rs6507054 | T | C | -0.00928 | 0.001393 | 0.00086 | 0.001422 | 0.020448 | 0.015482 | 0.032346 | 0.018195 | 0.061357 | 0.044269 |
| rs6530737 | A | G | 0.009019 | 0.001437 | 0.001399 | 0.001468 | 0.00211 | 0.016 | 0.019136 | 0.018761 | -0.0495 | 0.046496 |
| rs6548220 | A | G | 0.009358 | 0.001483 | 0.005241 | 0.001517 | -0.006 | 0.016562 | 0.002695 | 0.019509 | -0.00281 | 0.047881 |
| rs6575340 | G | A | -0.01332 | 0.00143 | -0.00569 | 0.00146 | 0.016187 | 0.015859 | 0.034516 | 0.018598 | -0.01452 | 0.045362 |
| rs6577497 | A | T | -0.0022 | 0.001403 | 0.008072 | 0.001434 | -0.0238 | 0.016037 | -0.01654 | 0.018952 | -0.07713 | 0.04651 |
| rs661878 | A | G | 0.008602 | 0.002018 | 0.013754 | 0.002061 | -0.03712 | 0.022055 | -0.0379 | 0.026732 | -0.04418 | 0.066267 |
| rs66460909 | G | A | 0.015693 | 0.001749 | 0.005274 | 0.001785 | 0.042484 | 0.019927 | 0.046417 | 0.023632 | 0.064916 | 0.058044 |
| rs6669341 | A | G | 0.010685 | 0.001387 | 0.006279 | 0.001418 | 0.002777 | 0.015504 | 0.005711 | 0.01824 | 0.03182 | 0.044282 |
| rs6679458 | G | T | -0.01156 | 0.00139 | -0.0067 | 0.001421 | -0.03559 | 0.015703 | -0.02993 | 0.018547 | -0.07275 | 0.045819 |
| rs67257872 | A | G | 0.010598 | 0.001379 | 0.007019 | 0.001409 | 0.019034 | 0.015322 | -0.00489 | 0.018632 | 0.027183 | 0.046805 |
| rs6752979 | G | A | -0.00896 | 0.00147 | -0.00367 | 0.001503 | 0.00539 | 0.016348 | -0.009 | 0.019194 | -0.01224 | 0.046886 |
| rs67603370 | G | A | -0.00046 | 0.002629 | -0.01583 | 0.002684 | 0.028218 | 0.032116 | 0.022033 | 0.03825 | -0.0674 | 0.097373 |
| rs6761463 | G | C | 0.012581 | 0.001858 | 0.003613 | 0.0019 | 0.023735 | 0.02047 | 0.025751 | 0.024045 | 0.034537 | 0.058549 |
| rs67913249 | C | G | 0.009346 | 0.00145 | 0.003071 | 0.001482 | 0.024422 | 0.016445 | 0.021173 | 0.019401 | 0.035193 | 0.047605 |
| rs680071 | T | C | -0.01174 | 0.002114 | -0.00614 | 0.00216 | -0.00928 | 0.024329 | -0.0436 | 0.030017 | 0.059682 | 0.072738 |
| rs6843852 | C | T | -0.00872 | 0.001371 | -0.00477 | 0.001401 | 0.003079 | 0.015308 | -0.00779 | 0.01803 | 0.04052 | 0.043916 |
| rs686431 | C | T | -0.01476 | 0.005089 | -0.03017 | 0.0052 | -0.0575 | 0.056041 | -0.14475 | 0.064214 | 0.019112 | 0.165694 |
| rs6870983 | C | T | 0.013797 | 0.001673 | 0.009701 | 0.00171 | 0.03216 | 0.018554 | 0.045447 | 0.021922 | -0.01602 | 0.052375 |
| rs6938973 | T | C | -0.01181 | 0.001401 | 0.005899 | 0.001431 | -0.00724 | 0.015566 | -0.00694 | 0.018297 | -0.0454 | 0.044508 |
| rs6950388 | G | A | -0.00945 | 0.001698 | 0.001148 | 0.001735 | 0.005307 | 0.019957 | 0.019477 | 0.023681 | 0.000958 | 0.058024 |
| rs6979832 | A | G | -0.0033 | 0.00138 | -0.00942 | 0.001409 | 0.01098 | 0.01544 | 0.001752 | 0.018236 | 0.049828 | 0.044239 |
| rs698147 | A | G | 0.00878 | 0.001377 | 0.001409 | 0.001407 | -0.01253 | 0.015604 | -0.0145 | 0.018421 | -0.08954 | 0.045447 |
| rs7012648 | G | A | -0.00685 | 0.001396 | -0.00974 | 0.001426 | 0.005048 | 0.015649 | 0.018244 | 0.018428 | 0.042128 | 0.044816 |
| rs7020196 | C | T | 0.007789 | 0.001413 | 0.000552 | 0.001444 | -0.01422 | 0.015875 | -0.00397 | 0.018679 | 0.044856 | 0.045888 |
| rs7020564 | A | T | 0.004974 | 0.001521 | 0.01002 | 0.001553 | -0.00243 | 0.016968 | -0.00729 | 0.01999 | -0.00549 | 0.04854 |
| rs7038966 | C | T | -0.00948 | 0.001401 | -0.00365 | 0.001431 | -0.01409 | 0.015599 | -0.02036 | 0.01835 | -0.06191 | 0.044796 |
| rs7084503 | T | C | 0.004868 | 0.001378 | 0.012897 | 0.001408 | 0.009905 | 0.015533 | 0.019352 | 0.018353 | 0.032384 | 0.044748 |
| rs7102934 | T | C | -0.00908 | 0.001494 | -0.00507 | 0.001526 | -0.02321 | 0.016568 | -0.03444 | 0.020185 | -0.01152 | 0.050844 |
| rs7103389 | T | C | -0.00946 | 0.001423 | -0.00453 | 0.001453 | 0.004679 | 0.015956 | 0.004322 | 0.019411 | 0.001915 | 0.04868 |
| rs7132908 | G | A | -0.01862 | 0.00141 | -0.03126 | 0.00144 | -0.02668 | 0.015685 | -0.02854 | 0.018439 | -0.00543 | 0.045537 |
| rs7145882 | T | C | 0.01146 | 0.001445 | 0.008188 | 0.001476 | -0.00431 | 0.015985 | 0.008896 | 0.018765 | -0.03331 | 0.046043 |
| rs71495049 | G | A | -0.01707 | 0.002478 | -0.00035 | 0.002532 | -0.03303 | 0.027445 | -0.03546 | 0.03216 | -0.00177 | 0.077926 |
| rs7182917 | T | C | 0.008737 | 0.001385 | 0.002246 | 0.001414 | 0.016155 | 0.015306 | 0.016895 | 0.018008 | 0.050387 | 0.043867 |
| rs7206608 | C | G | -0.00979 | 0.001469 | 4.55E-05 | 0.0015 | 0.006963 | 0.016308 | 0.007291 | 0.019158 | 0.037758 | 0.046981 |
| rs7239114 | G | A | -0.00644 | 0.001388 | -0.0135 | 0.001417 | -0.01879 | 0.015767 | -0.01617 | 0.018668 | -0.08925 | 0.045881 |
| rs724623 | A | C | 0.010244 | 0.001374 | 0.004373 | 0.001403 | -0.0358 | 0.015267 | -0.03806 | 0.017969 | 0.022008 | 0.043773 |
| rs7264802 | A | G | -0.00981 | 0.001591 | -0.00222 | 0.001624 | -0.00017 | 0.017951 | -0.00566 | 0.021123 | -0.02265 | 0.05149 |
| rs72753485 | G | C | -0.0156 | 0.002498 | -0.00368 | 0.002552 | -0.04561 | 0.029951 | -0.01395 | 0.035669 | -0.12215 | 0.085803 |
| rs72755233 | G | A | -0.00968 | 0.002179 | -0.01763 | 0.002225 | 0.006619 | 0.029182 | 0.009547 | 0.034922 | -0.0298 | 0.084747 |
| rs72892910 | G | T | -0.02528 | 0.001818 | -0.02466 | 0.001858 | -0.03334 | 0.019896 | -0.01516 | 0.023367 | -0.06363 | 0.057017 |
| rs72910629 | A | G | -0.01325 | 0.002011 | 0.00226 | 0.002055 | -0.04103 | 0.022173 | -0.03426 | 0.025921 | 0.009617 | 0.064419 |
| rs72917533 | T | C | 0.011462 | 0.001764 | 0.004664 | 0.001804 | -0.00386 | 0.021635 | 0.027995 | 0.025908 | 0.025527 | 0.065552 |
| rs72976986 | G | A | 0.013851 | 0.001766 | 0.000631 | 0.001803 | 0.02556 | 0.020444 | 0.029861 | 0.024148 | 0.077258 | 0.060053 |
| rs73026723 | C | T | 0.014068 | 0.0019 | 0.009485 | 0.00194 | -0.06269 | 0.021064 | -0.06346 | 0.024753 | -0.08705 | 0.059711 |
| rs7305424 | A | T | -0.00388 | 0.001453 | -0.01039 | 0.001483 | -0.00738 | 0.016431 | -0.01472 | 0.019381 | -0.06671 | 0.047808 |
| rs7306710 | T | C | 0.000735 | 0.001381 | 0.009966 | 0.001411 | -0.01196 | 0.015391 | -0.01188 | 0.0181 | 0.045643 | 0.04404 |
| rs7321285 | A | C | 0.011348 | 0.001719 | 0.001461 | 0.001756 | 0.02754 | 0.018894 | 0.025625 | 0.022161 | 0.052552 | 0.053666 |
| rs73213484 | A | T | 0.014638 | 0.001971 | 0.001243 | 0.002014 | 0.018284 | 0.021677 | 0.038606 | 0.025526 | -0.04005 | 0.061188 |
| rs7331420 | G | A | 0.009142 | 0.001526 | -0.00076 | 0.001558 | 0.009432 | 0.016738 | 0.025676 | 0.019718 | -0.04489 | 0.047323 |
| rs7354849 | A | G | -0.00426 | 0.00138 | -0.00817 | 0.00141 | -0.0017 | 0.016076 | -0.0202 | 0.019064 | 0.097818 | 0.047225 |
| rs7355953 | T | C | -0.01063 | 0.001671 | -0.01516 | 0.001709 | 0.028284 | 0.018617 | 0.012025 | 0.021861 | -0.01827 | 0.052715 |
| rs7424120 | C | T | 0.014201 | 0.001402 | 0.003979 | 0.001433 | 0.025869 | 0.015819 | 0.031321 | 0.018486 | 0.047151 | 0.045774 |
| rs7424771 | G | A | -0.00231 | 0.001376 | 0.00893 | 0.001407 | 0.005389 | 0.015378 | -0.01177 | 0.018056 | -0.02888 | 0.043947 |
| rs7439324 | C | T | 0.007527 | 0.001862 | 0.010771 | 0.001903 | 0.003931 | 0.020558 | 0.001638 | 0.024172 | 0.010207 | 0.059344 |
| rs7453694 | C | T | -0.00979 | 0.001521 | -0.00598 | 0.001554 | 0.021935 | 0.016464 | 0.036966 | 0.019334 | 0.036052 | 0.047327 |
| rs7498044 | G | A | 0.009952 | 0.001681 | -0.00032 | 0.001716 | -0.00098 | 0.019042 | -0.00246 | 0.022481 | -0.05799 | 0.054327 |
| rs7498665 | A | G | -0.01728 | 0.001401 | -0.01344 | 0.001431 | -0.05447 | 0.015455 | -0.06293 | 0.018137 | -0.0191 | 0.044324 |
| rs75001243 | C | T | -0.0068 | 0.001429 | -0.01373 | 0.00146 | -0.00386 | 0.016113 | -0.00085 | 0.018859 | -0.0808 | 0.046223 |
| rs7503580 | C | T | -0.00488 | 0.001893 | -0.01112 | 0.001933 | -0.00964 | 0.021714 | -0.00733 | 0.025649 | 0.014495 | 0.067361 |
| rs75387636 | G | A | -0.00746 | 0.003435 | -0.02122 | 0.00351 | 0.04714 | 0.038149 | 0.059006 | 0.04518 | -0.04204 | 0.106931 |
| rs7549358 | G | C | 0.00883 | 0.001429 | 0.003619 | 0.001461 | 0.008914 | 0.015935 | -0.0033 | 0.018766 | 0.050542 | 0.045225 |
| rs7550711 | C | T | -0.04264 | 0.004316 | -0.04841 | 0.004412 | -0.05744 | 0.044312 | -0.05438 | 0.051893 | -0.09731 | 0.122788 |
| rs7565437 | T | C | 0.002007 | 0.001394 | 0.008183 | 0.001426 | 0.02643 | 0.015785 | 0.024175 | 0.018604 | 0.070768 | 0.045792 |
| rs7606059 | T | C | -0.00405 | 0.001463 | -0.01051 | 0.001496 | 0.025759 | 0.016277 | 0.030595 | 0.018954 | 0.00864 | 0.047383 |
| rs7619139 | T | A | -0.00884 | 0.001395 | -0.0098 | 0.001427 | -0.00647 | 0.015992 | -0.01647 | 0.018936 | -0.04823 | 0.046953 |
| rs7656673 | A | G | -0.00828 | 0.001398 | -0.01156 | 0.001429 | -0.01627 | 0.015524 | -0.00821 | 0.018285 | -0.05034 | 0.044259 |
| rs76702514 | C | G | 0.010137 | 0.001689 | 0.003911 | 0.001727 | 0.002662 | 0.018886 | 0.012195 | 0.022236 | -0.01436 | 0.054562 |
| rs7672 | C | G | 0.004686 | 0.001527 | 0.009004 | 0.00156 | 0.019891 | 0.017075 | 0.010789 | 0.020112 | 0.02813 | 0.048751 |
| rs7719067 | A | G | 0.00931 | 0.001384 | 0.013452 | 0.001414 | 0.001823 | 0.01539 | 0.004136 | 0.018099 | 0.009988 | 0.044242 |
| rs7749708 | C | T | -0.00998 | 0.001509 | -0.00469 | 0.001541 | -0.01784 | 0.016604 | -0.01313 | 0.01948 | -0.02 | 0.047499 |
| rs7753558 | C | A | 0.005703 | 0.001431 | 0.009088 | 0.001463 | -0.01267 | 0.015983 | -0.0114 | 0.018876 | 0.046074 | 0.045396 |
| rs77960 | G | A | -0.00624 | 0.001461 | 0.010529 | 0.001493 | -0.0186 | 0.016189 | -0.02849 | 0.019024 | 0.02702 | 0.04677 |
| rs77976727 | C | T | -0.00744 | 0.002358 | -0.0151 | 0.002409 | -0.05784 | 0.026824 | -0.00849 | 0.031653 | -0.21166 | 0.075277 |
| rs7808296 | C | T | -0.00255 | 0.001476 | -0.01004 | 0.001508 | -0.03298 | 0.016689 | -0.05218 | 0.019654 | -0.02195 | 0.048391 |
| rs7869098 | T | G | 0.006361 | 0.001377 | 0.009843 | 0.001406 | 0.004831 | 0.015609 | 0.009148 | 0.018432 | -0.00019 | 0.045111 |
| rs788858 | A | G | 0.000576 | 0.00151 | 0.012289 | 0.001543 | 0.023762 | 0.017174 | 0.028826 | 0.020306 | 0.071537 | 0.049869 |
| rs78886584 | A | G | -0.00849 | 0.001385 | -0.00152 | 0.001416 | -0.02435 | 0.017951 | -0.02168 | 0.021018 | -0.06519 | 0.048697 |
| rs78907487 | A | C | -0.00677 | 0.001937 | -0.01237 | 0.001977 | 0.00573 | 0.021553 | 0.006685 | 0.02544 | -0.12047 | 0.059368 |
| rs7893571 | G | T | -0.01018 | 0.001457 | -0.00193 | 0.001489 | -0.00228 | 0.016549 | -0.00712 | 0.01956 | 0.048836 | 0.047527 |
| rs7924036 | G | T | 0.009634 | 0.001372 | 0.0041 | 0.001401 | 0.000448 | 0.015167 | 0.007297 | 0.017836 | 0.003294 | 0.043402 |
| rs7925100 | G | A | -0.00917 | 0.001403 | -0.00313 | 0.001433 | -0.00021 | 0.015546 | 0.005478 | 0.018907 | -0.03374 | 0.046897 |
| rs7931626 | C | T | 0.005271 | 0.001376 | 0.009703 | 0.001405 | 0.011698 | 0.015527 | 0.00399 | 0.019005 | 0.008763 | 0.048027 |
| rs7958241 | A | G | -0.00499 | 0.001445 | -0.01306 | 0.001476 | -0.03132 | 0.015995 | -0.03775 | 0.01875 | -0.03322 | 0.045827 |
| rs796915 | C | G | -0.00737 | 0.001491 | -0.01291 | 0.001523 | -0.01088 | 0.016725 | -0.02184 | 0.019703 | 0.046449 | 0.047624 |
| rs80082351 | A | G | 0.018501 | 0.002847 | 0.00749 | 0.002911 | -0.03616 | 0.032541 | -0.03593 | 0.038122 | -0.0159 | 0.097289 |
| rs80082536 | A | G | -0.01289 | 0.002125 | -0.00715 | 0.002173 | -0.02223 | 0.025697 | -0.04986 | 0.030311 | 0.049508 | 0.075396 |
| rs80236973 | C | T | 0.012159 | 0.002008 | 9.83E-05 | 0.002054 | 0.026261 | 0.02361 | 0.027001 | 0.02808 | 0.034961 | 0.069342 |
| rs8030456 | C | T | 0.018343 | 0.001636 | 0.021314 | 0.001671 | 0.050917 | 0.018309 | 0.038646 | 0.021554 | 0.065644 | 0.052702 |
| rs8038574 | T | C | 0.009074 | 0.001451 | 0.004846 | 0.001481 | 0.041017 | 0.015935 | 0.041321 | 0.018676 | -0.02737 | 0.046009 |
| rs8089514 | T | A | -0.00817 | 0.001439 | -0.00732 | 0.00147 | -0.01824 | 0.016417 | -0.01375 | 0.019318 | 0.043153 | 0.047632 |
| rs8117463 | G | A | 0.005189 | 0.001469 | 0.0089 | 0.001499 | 0.00177 | 0.016774 | 0.014842 | 0.019824 | -0.03507 | 0.048444 |
| rs8124896 | T | C | -0.01317 | 0.002284 | -0.00577 | 0.002331 | -0.03317 | 0.02521 | -0.01954 | 0.029853 | -0.06829 | 0.071856 |
| rs8134638 | T | C | -0.0078 | 0.00142 | 0.000768 | 0.001449 | 0.017018 | 0.015701 | 0.013087 | 0.018425 | 0.009247 | 0.045058 |
| rs815163 | T | C | 0.010754 | 0.001378 | 0.005836 | 0.001409 | -0.01272 | 0.015562 | -0.01078 | 0.018353 | 0.067458 | 0.045103 |
| rs818898 | A | G | 0.006192 | 0.00149 | 0.012067 | 0.001522 | 0.0497 | 0.016427 | 0.05726 | 0.019284 | 0.014156 | 0.046862 |
| rs8192675 | T | C | -0.0117 | 0.001509 | -0.00077 | 0.001543 | -0.03372 | 0.016874 | -0.04981 | 0.019865 | 0.02039 | 0.049112 |
| rs824207 | A | G | -0.00221 | 0.001375 | -0.00931 | 0.001404 | -0.00169 | 0.017379 | -0.02224 | 0.020986 | 0.04565 | 0.053459 |
| rs827803 | G | T | 0.007866 | 0.001381 | 0.00581 | 0.001412 | 0.026122 | 0.015364 | 0.017921 | 0.018052 | -0.02851 | 0.044055 |
| rs836179 | A | G | 0.002372 | 0.001421 | 0.009805 | 0.001451 | 0.005549 | 0.015972 | 0.000369 | 0.018824 | -0.01937 | 0.046154 |
| rs862320 | C | T | 0.014399 | 0.001396 | 0.006193 | 0.001426 | 0.032768 | 0.015416 | 0.023245 | 0.018108 | 0.083137 | 0.044307 |
| rs868784 | G | A | 0.007872 | 0.001417 | 0.002275 | 0.001447 | 0.001964 | 0.015927 | 0.012799 | 0.01937 | 0.029762 | 0.048995 |
| rs869400 | T | G | -0.01831 | 0.001766 | -0.00922 | 0.001806 | -0.01706 | 0.020146 | -0.01156 | 0.023703 | -0.03081 | 0.058295 |
| rs884152 | G | T | -0.00332 | 0.001432 | -0.00851 | 0.001463 | 0.000753 | 0.016123 | -0.00658 | 0.019015 | 0.038365 | 0.046433 |
| rs9260164 | C | T | -0.00986 | 0.00158 | -0.01517 | 0.001614 | 0.029771 | 0.018207 | 0.037359 | 0.021594 | 0.052971 | 0.052596 |
| rs9265968 | A | T | -0.00751 | 0.002065 | -0.02013 | 0.00211 | 0.091476 | 0.023213 | 0.098889 | 0.027511 | 0.048794 | 0.066513 |
| rs9291816 | C | T | 0.007976 | 0.001466 | 0.013379 | 0.001498 | 0.018187 | 0.016805 | 0.01669 | 0.019908 | 0.051682 | 0.049365 |
| rs9366863 | T | C | 0.017384 | 0.001457 | 0.007544 | 0.001489 | -0.00194 | 0.016143 | -0.00024 | 0.019007 | 0.066334 | 0.04592 |
| rs9438393 | A | G | 0.005266 | 0.001391 | 0.010349 | 0.001422 | 0.029299 | 0.015444 | 0.030507 | 0.018178 | 0.00838 | 0.043897 |
| rs9477762 | A | T | -0.02019 | 0.003052 | 0.000272 | 0.003119 | 0.032859 | 0.034331 | 0.037336 | 0.040253 | -0.06757 | 0.09892 |
| rs9515446 | A | G | -0.00969 | 0.00138 | -0.00046 | 0.00141 | 0.004304 | 0.015256 | -0.00555 | 0.017893 | -0.01827 | 0.043596 |
| rs9522180 | C | T | 0.009236 | 0.001382 | 0.00155 | 0.001412 | -0.01294 | 0.015615 | -0.01112 | 0.018427 | 0.048203 | 0.045336 |
| rs9529148 | G | A | -0.008 | 0.001424 | -0.00028 | 0.001454 | 0.021386 | 0.015824 | 0.033952 | 0.018641 | -0.00267 | 0.045583 |
| rs957512 | T | C | 0.008797 | 0.001463 | 0.010341 | 0.001494 | 0.000734 | 0.016166 | -0.01014 | 0.018988 | 0.023741 | 0.046938 |
| rs9579775 | A | C | -0.01465 | 0.002084 | -0.00468 | 0.002129 | -0.04343 | 0.028199 | -0.04257 | 0.033734 | 0.058814 | 0.080148 |
| rs9603697 | C | T | -0.00853 | 0.001465 | -0.01296 | 0.001496 | -0.02247 | 0.016288 | -0.03965 | 0.019171 | -0.07879 | 0.0467 |
| rs9610387 | G | A | 0.007887 | 0.002457 | 0.013799 | 0.002508 | -0.03605 | 0.028886 | -0.01525 | 0.034306 | -0.10408 | 0.083217 |
| rs9615723 | C | T | 0.007715 | 0.001403 | 0.002261 | 0.001432 | 0.012005 | 0.016236 | 0.015654 | 0.019159 | -0.03249 | 0.047279 |
| rs9652090 | G | T | -0.00371 | 0.001386 | -0.00833 | 0.001416 | -0.00776 | 0.015715 | -0.02059 | 0.018537 | 0.015036 | 0.045627 |
| rs9673839 | A | G | -0.0083 | 0.001379 | -0.0032 | 0.001408 | -0.02097 | 0.015353 | -0.01934 | 0.018058 | -0.00789 | 0.043994 |
| rs9788550 | G | C | 0.013787 | 0.001596 | 0.007681 | 0.00163 | 0.019984 | 0.017803 | 0.032797 | 0.020972 | -0.03584 | 0.051011 |
| rs9843653 | T | C | -0.01755 | 0.001371 | -0.00407 | 0.001402 | 0.006886 | 0.015239 | -0.00145 | 0.017954 | 0.020732 | 0.043311 |
| rs9888533 | C | T | -0.00765 | 0.001399 | -0.00115 | 0.001429 | 0.001291 | 0.015999 | -0.00026 | 0.018876 | -0.01574 | 0.045773 |

**Supplementary table 2: Genetically determined effect estimates from univariable Mendelian randomization analysis of the effects of child and adult body size on endometrial cancer outcomes**

| **Exposure** | **Outcome** | **Method** | **Number of SNPs** | **Odds ratio** | **Lower 95% confidence interval of odds ratio** | **Upper 95% confidence interval of odds ratio** | **P value** |
| --- | --- | --- | --- | --- | --- | --- | --- |
| Adult body size | Endometrial cancer | MR Egger | 288 | 3.26 | 1.98 | 5.36 | 5.12E-06 |
| Adult body size | Endometrial cancer | Weighted median | 288 | 2.75 | 2.05 | 3.71 | 2.38E-11 |
| Adult body size | Endometrial cancer | Inverse variance weighted | 288 | 2.42 | 2.00 | 2.91 | 1.9E-20 |
| Adult body size | Endometrioid histology | MR Egger | 288 | 3.02 | 1.71 | 5.35 | 0.000174 |
| Adult body size | Endometrioid histology | Weighted median | 288 | 2.65 | 1.90 | 3.70 | 9.57E-09 |
| Adult body size | Endometrioid histology | Inverse variance weighted | 288 | 2.56 | 2.07 | 3.17 | 6.27E-18 |
| Adult body size | Non-endometrioid histology | MR Egger | 288 | 3.89 | 1.11 | 13.63 | 0.034654 |
| Adult body size | Non-endometrioid histology | Weighted median | 288 | 1.57 | 0.69 | 3.58 | 0.287297 |
| Adult body size | Non-endometrioid histology | Inverse variance weighted | 288 | 1.66 | 1.04 | 2.66 | 0.034158 |
| Child body size | Endometrial cancer | MR Egger | 166 | 2.41 | 1.60 | 3.63 | 4.08E-05 |
| Child body size | Endometrial cancer | Weighted median | 166 | 2.17 | 1.58 | 2.98 | 1.48E-06 |
| Child body size | Endometrial cancer | Inverse variance weighted | 166 | 1.86 | 1.52 | 2.28 | 1.31E-09 |
| Child body size | Endometrioid histology | MR Egger | 166 | 2.51 | 1.56 | 4.05 | 0.00022 |
| Child body size | Endometrioid histology | Weighted median | 166 | 2.31 | 1.59 | 3.35 | 1.15E-05 |
| Child body size | Endometrioid histology | Inverse variance weighted | 166 | 2.09 | 1.66 | 2.65 | 6.45E-10 |
| Child body size | Non-endometrioid histology | MR Egger | 166 | 3.65 | 1.25 | 10.71 | 0.019457 |
| Child body size | Non-endometrioid histology | Weighted median | 166 | 1.90 | 0.76 | 4.73 | 0.168165 |
| Child body size | Non-endometrioid histology | Inverse variance weighted | 166 | 2.23 | 1.32 | 3.79 | 0.002894 |

**Supplementary table 3: Genetically determined effect estimates from multivariable Mendelian randomization analysis of the effects of child and adult body size on endometrial cancer outcomes**

| **Exposure** | **Outcome** | **Method** | **Odds ratio** | **Lower 95% confidence interval of odds ratio** | **Upper 95% confidence interval of odds ratio** | **P value** |
| --- | --- | --- | --- | --- | --- | --- |
| Adult body size | Endometrial cancer | IVW | 2.30 | 1.73 | 3.06 | 1.09E-08 |
| Child body size | Endometrial cancer | IVW | 1.11 | 0.80 | 1.52 | 0.532975 |
| Adult body size | Endometrioid histology | IVW | 2.28 | 1.65 | 1.65 | 6.9E-07 |
| Child body size | Endometrioid histology | IVW | 1.24 | 0.87 | 0.87 | 0.2381 |
| Adult body size | Non-endometrioid histology | IVW | 1.06 | 0.52 | 2.16 | 0.869997 |
| Child body size | Non-endometrioid histology | IVW | 2.26 | 1.02 | 4.99 | 0.043308 |
| Adult body size | Endometrial cancer | MR Egger | 2.31 | 1.69 | 3.16 | 1.67E-07 |
| Child body size | Endometrial cancer | MR Egger | 1.11 | 0.78 | 1.57 | 0.554236 |
| Adult body size | Endometrioid histology | MR Egger | 2.27 | 1.59 | 1.59 | 6.75E-06 |
| Child body size | Endometrioid histology | MR Egger | 1.24 | 0.83 | 0.83 | 0.292586 |
| Adult body size | Non-endometrioid histology | MR Egger | 1.02 | 0.47 | 2.23 | 0.956431 |
| Child body size | Non-endometrioid histology | MR Egger | 2.17 | 0.92 | 5.15 | 0.077685 |

**Supplementary table 4: Univariable Egger MR intercept and heterogeneity statistic**

| **Outcome** | **Exposure** | **MR Egger intercept** | **MR Egger intercept p value** | **MR Egger heterogeneity** | **IVW heterogeneity** |
| --- | --- | --- | --- | --- | --- |
| Endometrial cancer | Adult body size | -0.00406 | 0.206472 | Q=375 p value=0.0003 | Q=377 p value=0.00026 |
| Endometrioid histology | Adult body size | -0.00227 | 0.536871 | Q=354 p value=0.00362 | Q=355 p value=0.00388 |
| Non-endometrioid histology | Adult body size | -0.01157 | 0.153074 | Q=258 p value=0.88067 | Q=260 p value=0.87059 |
| Endometrial cancer | Child body size | -0.00446 | 0.159511 | Q=197 p value=0.0402 | Q=199 p value=0.03502 |
| Endometrioid histology | Child body size | -0.00316 | 0.392545 | Q=193 p value=0.05925 | Q=194 p value=0.06055 |
| Non-endometrioid histology | Child body size | -0.00858 | 0.304969 | Q=158 p value=0.62291 | Q=159 p value=0.62128 |

**Supplementary table 5: Multivariable Egger MR intercept and heterogeneity statistic**

| **Outcome** | **MR Egger intercept** | **MR Egger intercept p value** | **MR Egger heterogeneity** | **IVW heterogeneity** |
| --- | --- | --- | --- | --- |
| Endometrial cancer | -8.9E-05 | 0.960715 | Q=446 p=0.00001 | Q=446 p=0.00002 |
| Endometrioid histology | 0.000154 | 0.940141 | Q=415 p=0.00076 | Q=415 p=0.00086 |
| Non-endometrioid histology | 0.001038 | 0.816535 | Q=330 p=0.45909 | Q=330 p=0.47377 |
